# Volatile Organic Compound Chamber: A Novel Technology for Microbiological Volatile Interaction Assays

**DOI:** 10.3390/jof7040248

**Published:** 2021-03-25

**Authors:** Samuel Álvarez-García, Sara Mayo-Prieto, Guzmán Carro-Huerga, Álvaro Rodríguez-González, Óscar González-López, Santiago Gutiérrez, Pedro A. Casquero

**Affiliations:** 1 Grupo Universitario de Investigación en Ingeniería y Agricultura Sostenible (GUIIAS), Instituto de Medio Ambiente, Recursos Naturales y Biodiversidad, Universidad de León, Avenida Portugal 41, 24071 León, Spain; smayp@unileon.es (S.M.-P.); gcarh@unileon.es (G.C.-H.); alrog@unileon.es (Á.R.-G.); ogonl@unileon.es (Ó.G.-L.); pacasl@unileon.es (P.A.C.); 2 Grupo Universitario de Investigación en Ingeniería y Agricultura Sostenible (GUIIAS), Área deMicrobiología, Escuela de Ingeniería Agraria y Forestal, Universidad de León, Campus de Ponferrada, Avenida Astorga s/n, 24401 Ponferrada, Spain; s.gutierrez@unileon.es

**Keywords:** volatile organic compounds, VOCs, trichodiene, squalene, *Trichoderma*, *Fusarium*, *Rhizoctonia*, biocontrol, biological control

## Abstract

The interest in the study of microbiological interactions mediated by volatile organic compounds (VOCs) has steadily increased in the last few years. Nevertheless, most assays still rely on the use of non-specific materials. We present a new tool, the volatile organic compound chamber (VOC chamber), specifically designed to perform these experiments. The novel devices were tested using four *Trichoderma* strains against *Fusarium oxysporum* and *Rhizoctonia solani*. We demonstrate that VOC chambers provide higher sensitivity and selectivity between treatments and higher homogeneity of results than the traditional method. VOC chambers are also able to test both vented and non-vented conditions. We prove that ventilation plays a very important role regarding volatile interactions, up to the point that some growth-inhibitory effects observed in closed environments switch to promoting ones when tested in vented conditions. This promoting activity seems to be related to the accumulation of squalene by *T. harzianum*. The VOC chambers proved to be an easy, homogeneous, flexible, and repeatable method, able to better select microorganisms with high biocontrol activity and to guide the future identification of new bioactive VOCs and their role in microbial interactions.

## 1. Introduction

Volatile organic compounds (VOCs) are small organic molecules produced and emitted by a wide variety of organisms. These compounds often serve as mediators in communication, interaction, and/or competition between organisms [1]. Besides, they play a key role in the establishment of ecological communities and their interrelationships [2]. VOCs have been described as mediating animal, plant, and microbial communication. Regarding animals, VOCs have been widely studied as attractants and repellents [3,4]. Plant VOCs can function as herbivore deterrents [5], while volatile plant–plant interactions have been demonstrated to take part in defence activation against predators [6] and diseases [7]. Concerning microbe–plant interactions, Moisan et al. [8] demonstrated that fungal VOCs from both pathogenic and non-pathogenic strains promoted growth and flowering and modulated the induced systemic response (ISR) of the exposed plants. VOCs from some fungi enhanced plant defences, while others increased their susceptibility to attack by generalist caterpillars. A comprehensive review of the effects produced by bacterial VOCs in plant development can be seen in the work of Kai et al. [9].

Additionally, microbiological volatile interactions have proved to be highly relevant, leading to a wide range of effects, from growth inhibition to growth promotion [1,10]. They also exert morphological and physiological changes in the fungal and bacterial strains exposed to them [11]. Nevertheless, the importance of microbial volatile organic compounds (mVOCs) was understated for decades, both in terms of little research towards its comprehension and a lack of specific laboratory material for performing VOC assays [12,13]. Fortunately, the study of the role played by these molecules in the biological and ecological interactions between organisms has become increasingly important throughout the last few years [14,15,16,17,18].

Ecologically, rhizosphere microbes seem to regulate plant growth through VOC production, as demonstrated for bacteria [19], *Trichoderma viride* [20], or mycorrhizal fungi [21]. Moreover, fungal VOCs have been proven to affect plant root architecture [22,23] and even the ability of the plant to confront herbivores [24]. Besides, VOCs released by infected plants seem to prepare the defensive systems of nearby plants for the eventual attack of the pathogen [7]. Furthermore, Li et al. [2] revealed a collective volatile-mediated antagonism of soil bacteria against fungi. These authors proposed VOC production as an important bacterial strategy for defending occupied niches against invading fungi, stating that this mechanism may represent a major contribution to soil fungistasis.

In addition, fungal species have been described as producing a wide variety of VOCs: sesquiterpenes, furanes, alkenes, alcohols, phenols, aliphatic hydrocarbons, aldehydes, ketones, benzene derivatives, esters, etc. [9,12,25,26]. In this regard, sesquiterpenes seem to account for an important part of the differences between microorganisms [12]. Moreover, species from the fungal genera *Fusarium*, *Rhizoctonia*, and *Trichoderma* have been identified as among the most odoriferous ones using E-Nose and solid phase microextraction–gas chromatography/mass spectrometry (SPME–GC/MS) [12]. Furthermore, the nature and amount of the VOCs produced are influenced not only by the strain itself but also by external factors such as temperature, oxygen and nutrient availability, pH, incubation period, etc. [12].

*Trichoderma* is a well-described and widespread fungal genus used as an effective biological control agent (BCA). In this regard, some studies have focused on fungal interactions mediated by *Trichoderma*-produced VOCs [27]. For example, VOCs emitted by *Trichoderma* spp. showed a strong effect against plant pathogenic fungi such as *Botrytis cinerea* and *Alternaria brassicicola* [28]. In the same way, Li et al. [29] tested four *Trichoderma* species, confirming that all of them produced VOCs that inhibited *Fusarium oxysporum* growth, while Speckbacher et al. [30] explored the production of bioactive VOCs from *Trichoderma atroviride* in various conditions and in confrontation with *Rhizoctonia solani* and *F. oxysporum.*

Nowadays, three main methods are currently in use for VOC challenge assays: (i) divided Petri dishes, (ii) the plate-within-a-plate system, and (iii) the double dish set (DDS) method, also known as “two sealed base plates” or “sandwiched Petri plates”. The three of them possess certain advantages and limitations. Firstly, the divided Petri dishes are easy to manipulate and technically able to provide vented and non-vented conditions. Nevertheless, their structure poses a serious limitation in the available surface for microbial growth, and they are also highly prone to cross-contamination, as microorganisms, especially filamentous fungi, tend to overcome the dividing walls. Secondly, the plate-within-a-plate system is more difficult to set up, it requires more material and space and, most importantly, it presents important difficulties regarding management and data collection during the experiments. Finally, the two sealed base plates or double dish set (DDS) method [31] seems to be the preferred and most widely used setup for performing the in vitro assessment of volatile-mediated microbial interactions. Nevertheless, this technique presents several problems: risk of cross-contamination, low homogeneity and reproducibility due to incorrect or imperfect fitting, difficult and time-consuming assembling of the replicates, and the impossibility of varying the gas exchange rate with the exterior, producing a strong limitation on flexibility and on the ability to test different experimental conditions concerning ventilation. Thus, as stated by Alijani et al. [32], the results obtained with this technique are only applicable to airtight conditions. Moreover, gas exchange and oxygen availability play a significant role in microbial development [33] and microbial competition in biocontrol strategies [34]. For this reason, the outcome of the experiments performed with DDS or similar closed techniques may be strongly biased by this lack of ventilation, which could lay behind a significant part of the inhibitory activity detected in these experiments. Therefore, low oxygen concentrations may be distorting the final results, mistakenly assigned to the single bioactivity of VOCs.

Notwithstanding the aforementioned limitations, several authors have proposed the potential usefulness of VOCs for biotechnological and field applications, for example in biocontrol [14], as bio-fumigants [15], and other uses [35]. As these studies have been carried out in airtight environments, claims made regarding the usefulness of these microbial VOCs can only be accepted for the tested conditions until new trials are carried out. Surprisingly, it appears that almost no studies have been conducted so far to elucidate the effect that different ventilation produces on the microbiological interactions mediated by VOCs. Lo Cantore et al. [36] demonstrated that these interactions need to be approached in vitro by using non-airtight conditions. Their findings showed significant differences in the effect of bacterial volatiles using sealed and non-sealed divided Petri dishes. This need for the general testing of new conditions is further supported by the findings of Speckbacher et al. [30], who demonstrated the influence of light variation in the VOC production of *T. atroviride*.

In this regard, a few interesting modifications and alternative methods have already been proposed in the study of volatile interactions. For instance, Gershow et al. [3] presented a system used to deliver gaseous stimuli to study small animal navigation. Closer to the present study, some authors placed dialysis membranes between the plates to avoid cross-contamination when using the DDS method [37,38], or manually perforated plate lids to place these membranes on them [39]. Finally, Cernava et al. [40] developed a novel system for the fast screening and detection of bioactive microbial VOCs. Albeit definitely useful for their intended purposes, these last proposals still rely on non-specific materials, and they lack the ability to provide some important features to the experiments.

The VOC chambers presented here provide a standardized method for performing volatile assays, allowing for easier and faster use, better experimental homogeneity and replicability, and more flexibility regarding ventilation conditions. To prove the previous statements, we have compared the DDS method and two structurally different VOC chambers, namely non-vented and vented VOC chambers. The design of these new devices is derived from previous experiences in our research group. In this regard, we selected a wild type (WT) *T. harzianum* strain and three of its transformants that were previously developed, described, and their antifungal activity assessed in our group [41,42,43]. Furthermore, our group has also reported the attractive and repellent activity of these fungal strains against the insect pest *Acanthoscelides obtectus* [4]. In the present work, we confronted these BCAs against two phytopathogenic fungal strains isolated in our laboratory: *F. oxysporum* F3 and *R. solani* R43 [44].

Finally, we hypothesize that variations in ventilation produce significant effects on interactions between filamentous fungi mediated by VOCs, and that the herein presented VOC chambers are a more effective way to perform these kinds of experiments than the traditional methods. Hence, the main goals of this study are: (i) to present a novel technology specifically designed to perform microbiological VOC assays, (ii) to demonstrate its advantages (easy, homogeneous, flexible, and repeatable) in comparison to the traditional DDS method, and (iii) to further prove the importance of non-tightly closed environments in microbe–microbe interactions mediated by VOCs.

## 2. Materials and Methods

### 2.1. VOC Chamber: General Technical Description

**Figure 1:**
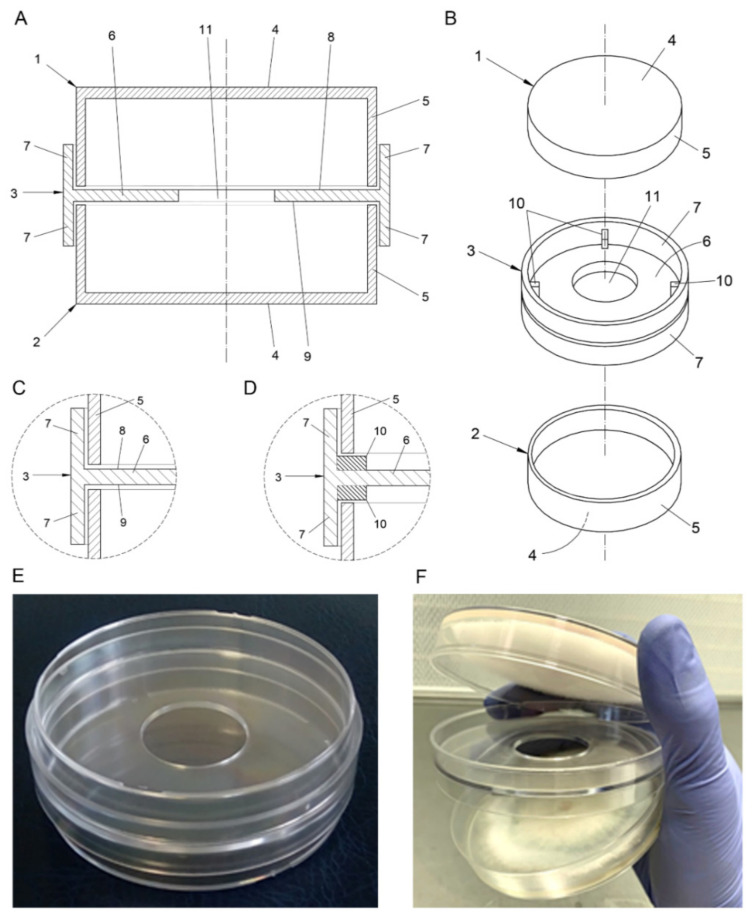
(**A**) Frontal cross-section, non-vented VOC chamber. (**B**) Explosive view, vented VOC chamber. (**C**) Detail of the union between the plates and the central piece in the non-vented VOC chambers. (**D**) Detail of the union between the plates and the central piece with flanges in the vented VOC chambers. 1: upper plate, 2: lower plate, 3: central piece, 4: upper and lower walls (plates), 5: perimeter wall (plates), 6: intermediate wall (central piece), 7: lateral walls (central piece), 8: upper face (intermediate wall), 9: lower face (intermediate wall), 10: ventilation flanges (vented VOC chambers), 11: central hole. (**E**) Closed empty VOC chamber. (**F**) Opened VOC chamber with *F. oxysporum* (upper plate) and *T. harzianum* (lower plate).

The novel device presented in this article is a culture chamber (Figure 1) specifically designed to perform microbiological competition and interaction assays mediated by VOCs. It has been patented with the number ES 2708899 B2 in the Spanish Office of Patents and Trademarks and has been submitted internationally with the PCT number PCT/ES2019/070475. The system is derived from the double dish set (DDS), also known as a “sandwiched Petri plates assay” or “two sealed base plate system” [31] and is comprised of three parts; two base plates equal to 90 mm standard Petri dishes (Figure 1, numbers 1 and 2), and a perforated central piece (Figure 1, number 3) that acts as a double lid, holding together and connecting the aforementioned plates facing each other. This central piece has a slightly larger diameter than the plates, and two lateral walls projecting upwards and downwards (Figure 1, number 7), thus being able to hold and house both plates. It has a central hole (Figure 1, number 11) that connects the headspaces of both plates, thus allowing VOCs to freely move from one culture to the other.

In order to perform mVOC interaction assays, the two base plates are used as regular Petri dish plates to pour culture media and grow microorganisms on. The lower plate is placed facing upwards, while the perforated central piece rests on the top of it as a lid would do. Finally, the upper plate rests on the upper horizontal face (Figure 1, number 8) of the central piece facing downwards. Therefore, both plates are firmly set facing each other and held by the central piece, which allows gas exchange between them through its hole.

The intermediate wall (Figure 1, number 6) of the central piece can present a flat surface on both sides like in a non-vented Petri dish (Figure 1D), thus limiting the gas exchange with the exterior and therefore allowing for a higher concentration of VOCs inside the chamber or, otherwise, can present a vented configuration (Figure 1C) with small flanges (Figure 1C, number 10) on both sides of the edge of the central piece’s intermediate wall, making both plates rest a little up from the bottom and thus allowing for a higher rate of gas exchange between the chamber and the exterior. This vented structure helps to reduce VOC concentration inside the chamber and ensures oxygen availability for the growing microorganisms, whereupon it can provide closer conditions to those present in some natural environments. This vented/non-vented configuration is one of the main new features provided by the VOC chamber, widening the range of experimental conditions previously available using the traditional DDS methodology.

Additionally, a membrane or filter (Figure S2, number 14) can be placed covering the hole of the central piece to avoid cross-contamination between cultures. Membranes or filters could potentially limit the crossing of certain active biological molecules, therefore acting as a way to sort the VOCs that can reach the opposing microorganisms. Neither filters nor membranes were used in the present study to cover the central hole.

Divided Petri dishes (Figure S1, number 15), while not tested in the present work, could be used to evaluate multilateral interactions between more than two microbiological strains. Further modifications of the prototype regarding the central piece or the plates and the dimensioning of the prototype are available in the supplementary materials.

### 2.2. VOC Chamber: Prototypes

The base plates used for the present research were obtained from 90 mm Petri dishes, while the central pieces were produced from polystyrene crystal by J.D. Catalán S.L. (Arganda del Rey, Madrid, Spain) using a plastic injection steel mold made specifically for this purpose. These central pieces (Figure 1, number 3) were 92 mm in diameter with a 30 mm central hole (Figure 1, number 11). Its peripheral walls (Figure 1, number 7) had a height of 15 mm and the plastic thickness was 1 mm in all parts of the piece.

Non-vented VOC chambers presented a central piece (Figure 1, number 3) with flat surfaces on both sides, while vented VOC chambers had 3 flanges (Figure 1, number 10) on both sides of the edge of the central piece’s horizontal wall. These flanges were 0.7 mm in height, leaving a space between the plates and the central piece, thus allowing for gas exchange between the chamber and the exterior, as explained in the previous section. Pictures of VOC chambers are presented in Figure 1E,F.

### 2.3. Microbial Strains and Culture Conditions

Two *Trichoderma* strains were used as parental strains: *Trichoderma harzianum* CECT 2413 (Spanish Type Culture Collection, Valencia, Spain) (T34 from now onwards), a well-established biocontrol agent, and *T. harzianum* E20, a transformant derived from T34 by silencing squalene epoxidase encoding gene *erg1*, which lead to the accumulation of squalene and produced low levels of ergosterol [41]. Expression of the *T. arundinaceum* tri5 gene in these strains resulted in the selection of two transformants: T34-5.27 and E20-5.7, respectively, which showed high levels of trichodiene production [42,43]. This last compound is the first and the only volatile intermediate in trichothecene biosynthesis and is considered a non-phytotoxic VOC. Furthermore, as a result of the *erg1* gene silencing, with the subsequent accumulation of farnesyl diphosphate (FDP) in the E20 strain, production of trichodiene was significantly higher in the strain E20-5.7 than in T34-5.27 [42,43].

Regarding phytopathogenic strains, *F. oxysporum* F3 and *R. solani* R43 were isolated from bean (*Phaseolus vulgaris* L.) fields belonging to the Protected Geographical Indication (PGI) “Alubia de La Bañeza-León” (Spain), and selected for their high virulence against this crop [44]. These strains are stored in the Pathogens and Antagonists Collection at the Pest and Diseases Diagnosis Laboratory (PALDPD, University of León, León, Spain). *Trichoderma* and *F. oxysporum* strains were preserved in 50% glycerol spore suspension at −80 °C, while *R. solani* was kept at 4 °C in slanted assay tubes with PDA (potato dextrose agar, Difco Becton Dickinson, Sparks, MD, USA) and sealed with Parafilm^®^. All cultures were activated by culturing on PDA at 25 °C.

### 2.4. In Vitro Evaluation of VOC-Mediated Interactions between R. solani R43 and Trichoderma Using DDS Method, Vented, and Non-Vented VOC Chambers

A “double dish set” (DDS) method was performed as described by Dennis and Webster (1971) with some modifications. 6 mm plugs from the fresh edge of 2-day-old *Trichoderma* colonies were placed on the centre of 90 mm Petri dishes containing 18 mL of PDA, and were left to grow for 48 h at 25 °C. After that, *R. solani* R43 6 mm plugs from the edge of 3-day-old colonies were inoculated on the centre of 18 mL PDA Petri dishes. The respective lids of both *Trichoderma* and *R. solani* R43 were immediately removed, setting the plates facing each other and sealing both with two layers of Parafilm^®^ (Merck KGaA, Darmstadt, Germany). *Trichoderma* strains were always placed on the lower plate, facing upwards, while the pathogen was grown on the upper plate, and, therefore, facing downwards. This technique creates a tightly closed chamber where VOCs concentrate and move freely to and from both strains.

Assays with non-vented and vented VOC chambers were carried out in the same manner, except for the use of non-vented and vented central pieces, respectively, to hold and connect both the upper and lower plates. Besides this, the non-vented VOC chambers were sealed with two layers of Parafilm^®^, while the vented ones were not.

**Figure 2:**
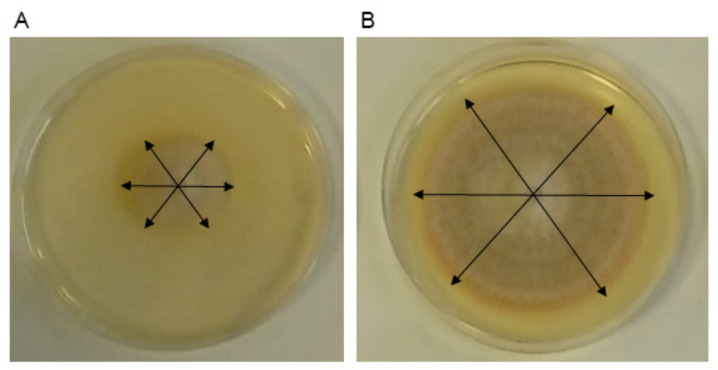
(**A**,**B**) *R. solani* R43 growth previously exposed to *T. harzianum* VOCs. The arrows represent the three diameters measured (in mm) per replicate. Their mean −6 (mm) represents the diameters used for growth comparisons and to calculate the percentage of inhibition (PI) for each treatment: PI = [(C − T) / C] × 100. Where C is the diameter of the control and T the diameter of the treatment.

Four repetitions were performed for each treatment and technique, and all of them were incubated at 25 °C. Control treatments were performed in the same way using PDA without *Trichoderma* in the lower plates. Colony diameters of *R. solani* R43 were measured in three different directions (Figure 2) with a ruler after 1, 2, 3, and 4 days after inoculation, considering the growth for each replicate as the mean of the aforementioned three measures (expressed in mm). Two sets of experiments were performed separately, both using T34, T34-5.27, E20, and E20-5.7 as biocontrol agents.

### 2.5. In Vitro Evaluation of VOC-Mediated Interactions between F. oxysporum F3 and Trichoderma Using the DDS Method, Vented, and Non-Vented VOC Chambers

The same protocol, as described in the previous section, was performed to evaluate the effect of *Trichoderma* spp. VOCs on *F. oxysporum* F3. Due to the slower rate of growth showed by this pathogen, plugs from 5-day-old *F. Oxysporum* F3 colonies were used in these assays, and data collection was carried out after 3, 5, and 7 days post-inoculation.

### 2.6. Data Treatment and Statistical Analysis

Microbial growth was considered as the mean diameter from each replicate, and 6 mm were subtracted from all measures to avoid distortions produced in the percentage of inhibition (PI) by the diameter of the plugs, as shown by Mutawila et al. [45]. PIs were calculated in relation to the control using the following equation proposed by Gotor-Vila et al. [46]: PI = [(C − T) / C ] × 100. Where C is the diameter of the control and T that of the treatment (Figure 2).

Microbial growth data (Diameter −6 mm) obtained from the VOC-mediated interaction assays were analysed with one-way analysis of variance (ANOVA, *p* ≤ 0.05, *p* ≤ 0.01 and *p* ≤ 0.001) after confirmation of the normality and equality of variances. Subsequently, *Trichoderma* treatments were contrasted between them and with the control within each technique/method (DDS, non-vented, and vented) using Tukey’s post hoc test (*p* ≤ 0.05, *p* ≤ 0.01, and *p* ≤ 0.001).

Moreover, the coefficient of variation (CV) or relative standard deviation (RSD) was calculated separately for each treatment and method to evaluate their precision and reproducibility. The equation being CV = σ/µ, in which σ represents the standard deviation and µ the mean for each treatment. The CV obtained were analysed with one-way analysis of variance (ANOVA, *p* ≤ 0.05) after confirmation of the normality and equality of variances and were contrasted between the three methods within *R. solani* R43 and *F. oxysporum* F3 confrontation assays using Tukey’s post hoc test (*p* ≤ 0.05). All statistical analyses were performed using SPSS v24.0 (IBM).

## 3. Results

### 3.1. Homogeneity and Replicability of the Results

In this work, we have evaluated the effects of VOCs produced by several Trichoderma spp. isolates on the development of two phytopathogenic fungi, and we have compared the results obtained by using the routine DDS method and the novel VOC chambers, both non-vented and vented versions, in order to elucidate their influence over the results.

The higher homogeneity provided by the new methodology is clearly shown by the lower coefficient of variation (CV) obtained in both *R. solani* R43 and *F. oxysporum* F3 assays (Table 1). In this regard, non-vented and vented VOC chambers presented a CV significantly lower than the results obtained with the DDS method, both using *R. solani* R43 as well as *F. oxysporum* F3. Additionally, there were no significant differences in CV between vented and non-vented VOC chambers. These results indicate that the use of the VOC chamber provides a higher degree of homogeneity and replicability than the traditional DDS method. The comparison of the results from experiment 1 and experiment 2 revealed significant differences between them, thus both were analysed and are presented separately (Table 1).

### 3.2. Volatile Activity of Trichoderma Against R. solani R43 and F. oxysporum F3. Statistical Differences in Growth Results Using the DDS Method, Non-Vented, and Vented VOC Chambers

**Figure 3:**
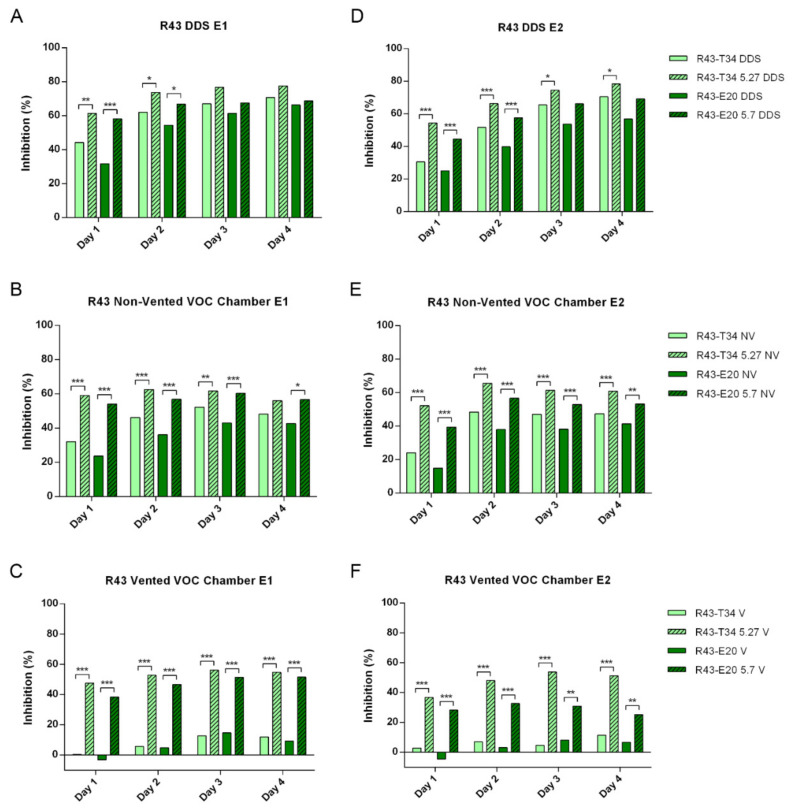
Inhibition percentages (PI) of *R. solani* R43 exposed to *T. harzianum* T34, T34-5.27, E20, and E20-5.7 VOCs after 1, 2, 3, and 4 days using (**A,D**) the DDS method (DDS, upper row), (**B,E**) the non-vented VOC chambers (NV, middle row), and (**C,F**) the vented VOC chambers (V, lower row). (**A–C**) Experiment 1, left column; (**D–F**) Experiment 2, right column. Statistical differences between each parental strain (T34 and E20) and their respective trichodiene-overproducing transformant (T34-5.27 and E20-5.7) were obtained from mycelial growth using ANOVA and Tukey’s post hoc test, and are represented between treatments within the same method and day by *** = *p* ≤ 0.001, ** = *p* ≤ 0.01, and * = *p* ≤ 0.05. No asterisks mean no significant differences. *n* = 4. Please note that the scales in the Y-axis of the figures representing vented VOC chambers (**C,F**) are compressed compared to the DDS (**A,D**) and non-vented ones (**B,E**), due to the presence of negative results.

**Figure 4:**
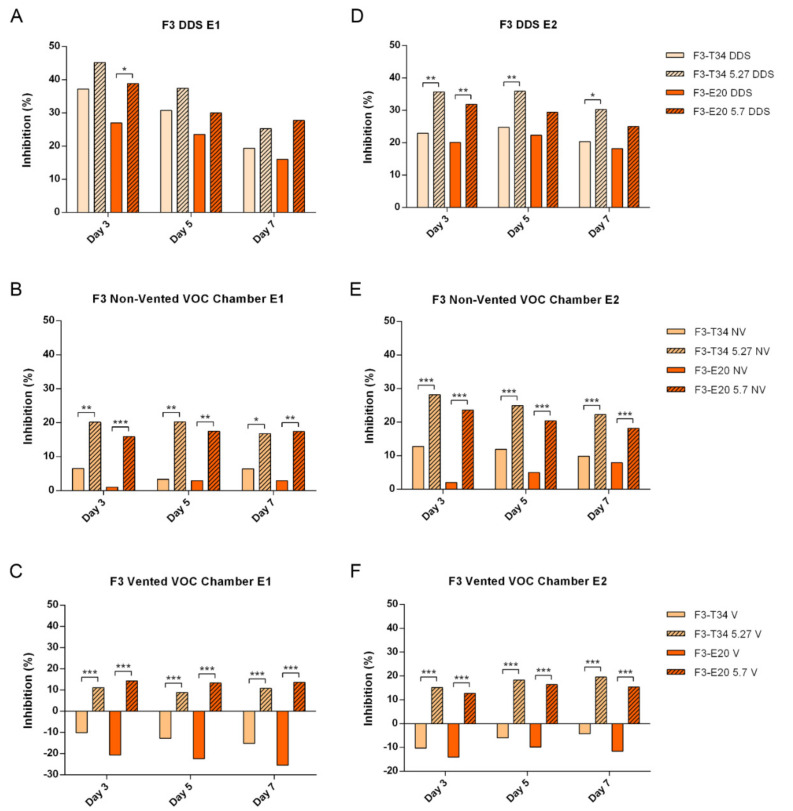
Inhibition percentages (PI) of *F. oxysporum* F3 exposed to *T. harzianum* T34, T34-5.27, E20, and E20-5.7 VOCs after 3, 5, and 7 days using (**A**,**D**) the DDS method (DDS, upper row), (**B**,**E**) the non-vented VOC chambers (NV, middle row), and (**C**,**F**) the vented VOC chambers (V, lower row). (**A**–**C**) Experiment 1, left column; (**D**–**F**) Experiment 2, right column. Statistical differences between each parental strain (T34 and E20) and their respective trichodiene-overproducing transformant (T34-5.27 and E20-5.7) were obtained from mycelial growth using ANOVA and Tukey’s post hoc test, and are represented between treatments within the same method and day by *** = *p* ≤ 0.001, ** = *p* ≤ 0.01, and * = *p* ≤ 0.05. No asterisks mean no significant differences. *n* = 4. Please note that the scales in the Y-axis of the figures representing vented VOC chambers (**C,F**) are compressed compared to the DDS (**A,D**) and non-vented ones (**B,E**), due to the presence of negative results.

When R43 growth was evaluated in confrontation with the different *T. harzianum* strains, the DDS method showed clear differences between the treatments and the control (*p* ≤ 0.001) and was also able to expose some statistical differences between trichodiene-overproducing strains (T34-5.27 and E20-5.7) and their parental strains (T34 and E20). Nevertheless, these differences were less significant than those observed using non-vented and vented VOC chambers and disappeared towards the last few days of the experiments (Figure 3A,D, Tables S1 and S2). For the same pathogenic strain, the non-vented VOC chambers rendered equally clearly significant differences between the treatments and the control (*p* ≤ 0.001) and, additionally, showed these differences between trichodiene-overproducing strains and their parental strains throughout the whole length of the experiment, mostly with *p* ≤ 0.001 (Figure 3B,E, Tables S1 and S2). Finally, the results obtained using the vented VOC chambers produced quite a different statistical output, indicating that, in these open conditions, the parental strains (T34 and E20) do not present significant inhibitory activity against *R. solani* R43 for the most part, while the trichodiene-overproducing strains retain their significant inhibitory activity (*p* ≤ 0.001) (Figure 3C,F, Tables S1 and S2). The vented VOC chambers showed statistical differences between trichodiene-producing strains and their parental strains up to day 4 in both experiments (*p* ≤ 0.001), except for the comparison between E20 and E20-5.7 during days 2 and 3 in experiment 2, which showed differences with *p* ≤ 0.01. Moreover, *F. oxysporum* F3 growth exposed to the different *T. harzianum* strains using the DDS method showed statistical differences between the *Trichoderma* treatments and the control (*p*≤ 0.01 and *p* ≤ 0.001). Nevertheless, it failed to show, in most cases, statistical differences between trichodiene-overproducing strains (T34-5.27 and E20-5.7) and their parental strains (T34 and E20). In experiment 1 these differences were only present between E20 and E20-5.7 on day 3 (*p* ≤ 0.05), while in experiment 2, they were also presented on day 3 comparing T34 and T34-5.27, but in both cases disappeared towards the last few days of the experiment (Figure 4A,D, Tables S3 and S4). For the same pathogenic strain, the non-vented VOC chambers rendered significant differences between the trichodiene-overproducing strains and the control (*p* ≤ 0.01 and *p* ≤ 0.001), but not so much between the parental strains and the control treatment, especially in the case of E20, which, in most cases, grouped with the control. Additionally, the results using the non-vented VOC chambers showed clear differences between trichodiene-overproducing strains and their parental strains throughout the whole length of the experiment, mostly with *p* ≤ 0.001 and *p* ≤ 0.01 (Figure 4B,E, Tables S3 and S4). Finally, the results obtained using the vented VOC chambers produced quite a different statistical output, indicating that in these open conditions the parental strains (T34 and E20) do not only not present significant inhibitory activity against *F. oxysporum* F3, but exert a significant promotion of its growth compared to the control (*p* ≤ 0.01 and *p* ≤ 0.001). On the contrary, the trichodiene-overproducing strains retain their significant inhibitory activity (*p* ≤ 0.05, and *p* ≤ 0.01 in experiment 1, and *p* ≤ 0.001 in experiment 2) (Figure 4C,F, Tables S3 and S4). The vented VOC chambers showed statistical differences between trichodiene-producing strains and their parental strains up to day 7 in all cases for both experiments (*p* ≤ 0.001).

### 3.3. Volatile Activity of Trichoderma Against R. solani R43 and F. oxysporum F3. Quantitative and Qualitative Differences in Percentage of Inhibition (PI) Using the DDS Method, Non-Vented, and Vented VOC Chambers

Firstly, it can be seen from Figure 3 that PI are consistently lower in vented conditions in comparison to the non-vented VOC chamber, and especially compared to the results obtained from the DDS treatments. In this regard, *R. solani* R43 exposed to the *Trichoderma* volatiles showed values of PI mostly between 50% and up to more than 78% using the DDS method (Figure 3A,D). These PI values were generally lower when using the non-vented VOC chambers, being between 32% and 65% (Figure 3B,E). As stated in the previous section, *T. harzianum* strains that overproduce trichodiene (T34-5.27 and E20-5.7) showed a significantly higher PI than their parental strains. Finally, the results obtained using the vented VOC chambers with *R. solani* R43 showed much lower PI values with maximums of around 56% exerted by T34-5.27 and 53% by E20-5.7, to mere 12% and 14% for T34 and E20, respectively (Figure 3C,F). On the other hand, *F. oxysporum* F3 exposed to *Trichoderma* volatiles showed a generally lower PI than that demonstrated against R43. When using the DDS method, all treatments significantly inhibited *F. oxysporum* F3 growth, with PI values between 16% and 25% for E20, to between 25% and 45% for T34 5.27 (Figure 4A,D). The results rendered by the use of non-vented VOC chambers demonstrated a lower inhibitory activity of the *Trichoderma* volatiles against *F. oxysporum* F3 than the DDS method. In this regard, the parental strains (T34 and E20) showed very low PI, albeit statistically significant in some cases compared to the control. Using the same method, the trichodiene-overproducing strains presented significantly higher PI than their parental ones, while still lower than the one they presented in DDS conditions by themselves (Figure 4B,E). Finally, using the vented VOC chambers, T34-5.27 and E20-5.7 presented lower, but still statistically significant, PI against *F. oxysporum* F3 compared to the control. By contrast, T34 and, especially, E20 showed important negative PI, indicating a completely opposite growth-promoting activity on *F. oxysporum* F3, being between −4% and −15% for T34 and from around −10% to −25% for E20 (Figure 4C,F). This increase in growth rate was statistically significant in all days and both experiments for E20 and for the most part when confronting *F. oxysporum* F3 to T34. All growths, PIs, and P values are represented in the Supplementary Tables S1, S2, S3, and S4. These results obtained comparing the three setups revealed that VOC’s effects, and the fungal responses to them, are strongly affected, both quantitatively and qualitatively, by the modification of the experimental conditions. Ventilation and gas exchange with the exterior seem to play a very important role in microbe–microbe volatile interactions.

### 3.4. Effects of erg1 Silencing and T. arundinaceum tri5 Expression on the Volatile Activity of T. harzianum T34 against R. solani R43 and F. oxysporum F3

**Figure 5:**
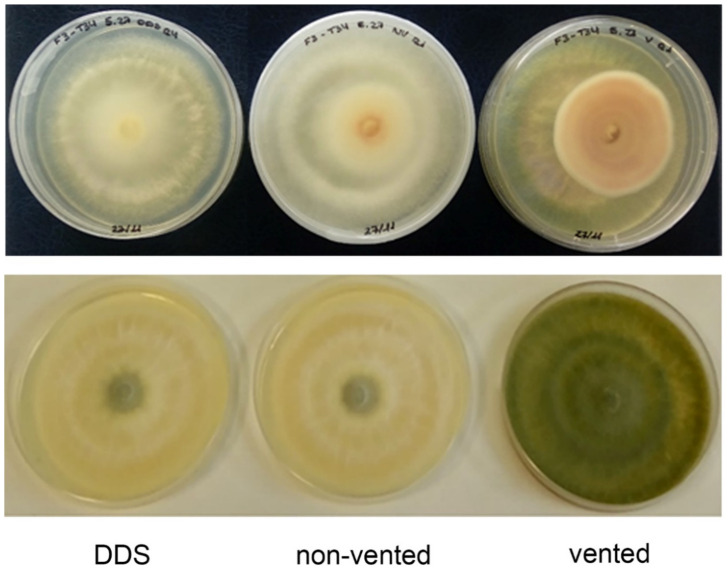
Fungal growth and mycelial aspect after 7 days in volatile assay. *F. oxysporum* F3 in confrontation with T34-5.27 (upper row). *T. harzianum* T34-5.27 after confrontation with *F. oxysporum* F3 (lower row). Both images show results from the DDS method (left column), non-vented VOC chamber (central column), and vented VOC chamber (right column). *F. oxysporum* F3 mycelium shows a faint growth, difficult to perceive, and a loss of coloration in DDS and non-vented VOC chambers, while *T. harzianum* shows thicker growth and more sporulation in vented conditions.

Regarding the activity of the VOCs produced by the wild type T34, results show a high inhibitory effect against *R. solani* R43 in both DDS and non-vented VOC chamber conditions (Figure 5). Nonetheless, as previously stated, using vented VOC chambers, the PI dropped dramatically, and its growth was not statistically different from the control treatment in most cases (Supplementary Tables ST1 and ST2). Additionally, this wild type strain showed a consistent growth inhibition activity against *F. oxysporum* F3 with DDS, and some inhibitory activity with non-vented VOC chambers, while demonstrating an opposite effect of growth promotion in vented conditions (Supplementary Tables S3 and S4). From this starting point, the results reveal a decrease in the inhibitory activity shown by the E20 strain compared to the WT. This decrease is consistent for *R. solani* R43 in all days and using any of the three techniques, but only statistically significant in some cases using DDS and especially non-vented VOC chambers (Tables S1 and S2). Regarding *F. oxysporum* F3, the DDS method failed to show statistical differences between T34 and E20, while non-vented VOC chambers were able to reveal them in some cases. However, using vented VOC chambers, the transformant E20 showed an increase in its growth-promoting activity on *F. oxysporum* F3 compared to WT T34 (Tables S3 and S4).

The results obtained from the T34-5.27 and E20-5.7 transformants revealed that the introduction of the *tri5* gene significantly increased the inhibitory effects of the overall VOCs produced by both strains. As stated, these effects were less significant when using the DDS method, which failed to find consistent statistical differences between the transformed strains and their parental ones. On the contrary, assays using non-vented and vented VOC chambers showed the relevant effect of this genetic modification, being not only able to increase the inhibitory capacity of the *Trichoderma* strains in non-vented VOC chambers but also producing an inversion of effects in vented ones, from significant growth promotion by T34 and E20 to significant growth inhibition by T34-5.27 and E20-5.7 (Figure 4, Tables S3 and S4).

### 3.5. Additional Effects Produced by the Used of DDS, Non-Vented and Vented VOC Chambers

Pathogens cultivated in DDS and, to a lesser extent, in non-vented VOC chambers presented a fainter growth, especially on the edges (Figure 5). This effect hindered data collection and was not observed when vented VOC chambers were used. Additionally, in the vented VOC chambers, the pathogens presented very clear compact edges that were easier to measure. This faint growth phenomenon was present in both pathogens but was stronger in *F. oxysporum* F3 than in *R. solani* R43 and was also observed in the DDS and non-vented controls. Moreover, not only the pathogens but also *Trichoderma* strains showed a fainter growth and less or no sporulation in DDS and non-vented conditions (Figure 5), indicating as well that their development is affected by the lack of gas exchange with the exterior. Moreover, *Trichoderma* volatiles produced a loss of pigmentation in *F. oxysporum* F3 mycelia. This effect was altered by the different ventilation setups, being non-existent in vented conditions and higher in non-vented and, especially, in DDS ones (Figure 5).

## 4. Discussion

As previously stated, regarding microbe–microbe VOC interactions, the DDS method [31] seems to be the preferred system in many recent studies [47]. A few interesting modifications and alternative methods have been proposed to date [37,39,40]. On the other hand, oxygen availability and its concentration play a key role in microbe development [33], and thus it must be of the utmost importance regarding microbial interactions. Besides, many of these ecological interactions take place in non-tightly closed environments. Hence, new experiments taking into account different ventilation conditions are needed. For that purpose, the novel VOC chambers described in this work were compared with the routine DDS method, evaluating the effects of VOCs produced by four *Trichoderma* strains against two phytopathogenic filamentous fungi: *R. solani* R43 and *F. oxysporum* F3.

Many previous studies using tightly closed routine methods, including the modifications stated before, reported important antimicrobial inhibitory activities exerted by VOCs, using bacteria [11,46,47], fungi [29,48,49], or yeast [50] as biocontrol agents. Our findings demonstrated an overall significantly higher inhibitory activity of VOCs from *Trichoderma* on *R. solani* than on *F. oxysporum*. These differences are consistent within all tested *Trichoderma* treatments and the three methodologies employed. Our findings are also in accordance with those described by Kashyap et al. [51], who reported inhibition percentages of around 60% when confronting *R. solani* to different *Trichoderma* strains, while Kai et al. [52] described *R. solani* inhibition between 80% and 99% when confronted to bacterial VOCs. In addition, Giorgio et al. (2015) tested different fungal pathogens against bacterial VOCs and reported *R. solani* to be one of the most inhibited fungi, while the two *F. oxysporum* strains evaluated appeared to be among the less inhibited ones. Moreover, as we report in this study, these authors also described a loss of pigmentation in *F. oxysporum* mycelia in the presence of microbial VOCs. However, our results indicate that *F. oxysporum* F3 does not suffer this loss in vented conditions. Interestingly, this alteration could be of significance to both metabolic aspects of the fungi, and also for some features related to the pathogen’s virulence and its antimicrobial capacity, as the pinkish-purple naphthoquinones produced by *F. oxysporum* were demonstrated to possess antimicrobial activity [53,54]. Moreover, a recent study evaluated the effects of the overproduction of trichodiene by *T. harzianum* on *Fusarium graminearum*, finding that it decreases its production of the mycotoxin deoxynivalenol [55].

### 4.1. Differences in Selectivity and Homogeneity Between Methods

The present findings highlight the capacity of the VOC chambers to reveal significant differences between treatments that were not shown up by the traditional DDS method. Therefore, VOC chambers demonstrated higher selectivity between treatments in almost all of the circumstances tested thus far, revealing new relevant information that could not be detected using the DDS method. This is a very important feature that will allow for better selecting those microorganisms with higher biocontrol potential in terms of VOC production, as well as to guide the future identification of new bioactive volatile compounds, and the investigation of their effects at molecular, genetic, and physiological levels. Therefore, the VOC chambers could become a very useful tool both for applied and basic research regarding volatile interactions. Furthermore, our study indicates that the coefficient of variation (CV) obtained from the data using non-vented and vented VOC chambers provides a higher degree of homogeneity than the DDS method.

### 4.2. Quantitative and Qualitative Effects of Different Ventilation Conditions

The results presented in this study comparing the three setups (DDS, non-vented, and vented VOC chambers) revealed that VOC’s effects and the fungal responses to them are strongly affected, both quantitatively and qualitatively, by the modification of the experimental conditions. Ventilation and gas exchange with the exterior indeed seem to play a very important role in fungus–fungus volatile interactions. Besides, the higher PI showed in DDS and non-vented assays appeared to come not only from a higher accumulation of toxic volatiles, but also from the significant reduction in oxygen in the headspace. This being supported by the fainter growth and physiological alterations presented by the fungi in airtight conditions. This effect should not be surprising, as oxygen concentration is of the utmost importance regarding microbial development [33], and could be exacerbated by the use of fast-growing biocontrol strains that would consume the available oxygen at a faster pace, like *Trichoderma* spp. This lack of oxygen could lie behind a significant part of the inhibitory activity detected in DDS and non-vented conditions in the present study, as well as of that reported previously in other studies using similar techniques. This could therefore jeopardize and distort the final results, mistakenly assigned to the single bioactivity of VOCs. Nevertheless, it should be taken into account that oxygen limitation can also induce microbial secondary metabolite formation, as reported by Clark et al. [56], and that effective competition for oxygen can be vital to controlling pathogens in certain conditions [34].

Furthermore, the present paper reports a surprising switch from inhibitory effect to growth-promoting effect produced by T34 and E20 VOCs on *F. oxysporum* F3 when using vented VOC chambers. This change could represent adaptive traits developed by the pathogenic and/or the biocontrol fungal strains in non-tightly closed natural environments, which, up until now, were concealed in vitro by the quasi-isolating characteristics of the DDS method. Promoting effects exerted by VOCs had already been reported in *Fusarium* spp. [57], but to our knowledge, this is the first time that ventilation conditions have been proven to produce qualitative changes from inhibitory to promoting effects using filamentous fungi as biocontrol agents against fungal pathogens. Interestingly, our findings are in contradiction with those obtained by Lo Cantore et al. [36], who used divided Petri dishes and reported an increase in the mycelia growth of *Pleurotus ostreatus* and *Pleurotus eryngii* when exposed to VOCs produced by *Pseudomonas tolaasii* in non-vented conditions, as opposed to an inhibitory effect in vented ones. This disparity of results may partially be due to the fast-growing rate of *Trichoderma* spp. in comparison to bacterial cultures, and therefore, faster oxygen consumption in the headspace. Albeit contradictory, both results pose a strong case towards the convenience of completing past and future VOC-mediated competition studies to assess the effect of different ventilation conditions on the microbial interactions mediated by VOCs. To summarize, three major findings indicate different effects of *Trichoderma* VOCs over the two pathogenic strains tested. Firstly, the overall significantly higher inhibitory activity of VOCs from *T. harzianum* on *R. solani* R43 than on *F. oxysporum* F3. Secondly, the general reduction of inhibitory activity in vented conditions compared to less open ones. Additionally, and thirdly, the most striking result obtained, the switch from inhibitory to growth-promoting effect of T34 and, especially, of E20 VOCs on *F. oxysporum* F3 in vented conditions.

All these findings may be rooted in ecological adaptations developed by both the pathogens and the *Trichoderma* strains, leading to complex microbiological interactions and the coevolution of the different fungal strains in their diverse natural environments. In this regard, DDSs and non-vented VOC chambers could be considered to represent environments where airflow and gas exchange face important limitations [32]. For instance, the conditions faced by microorganisms in some storing facilities [15,58] or the microbial interactions taking place in flooded areas or in soils with a clay texture. On the other hand, we believe that vented VOC chambers give very valuable information about the behaviour of microorganisms when exposed to volatile interactions in less tightly closed environments, as in soils with a sandy texture, aerial parts of the plant’s surface, and other open-air conditions. New studies should be conducted to elucidate how and why some microorganisms, as *F. oxysporum* F3 in our case, react in such different ways when facing VOCs from other microbial strains in vented and non-vented conditions, as well as to explain its link with the ecological interactions that might be explained by this behaviour.

### 4.3. Effects of the erg1 Silencing and tri5 Expression

Comparing to T34, the results using the E20 strain revealed a decrease in its inhibitory activity, being statistically significant in some cases. In fact, a slight promoting effect was observed for E20 in vented conditions, but without significant differences to the control. These findings suggest that the silencing of *erg1* and the subsequent reduction in ergosterol levels and the increase in squalene production may lead to lower production of toxic VOCs. It may indicate that the ergosterol route is implicated in the production of volatile compounds with antifungal activity, most likely of triterpene nature, but still unidentified. This is in agreement with the findings described previously where a reduction of bioactive activity was reported for the same transformants but in relation to soluble metabolites [41]. The high antifungal activity exerted by the wild type strain T34 on *R. solani* R43, and to a lesser extent on *F. oxysporum* F3, could derive from the production of some bioactive VOCs already described for *T. harzianum* and other species. In this regard, dimethyl disulfide and dimethyl trisulfide were identified by Elkahoui et al. [59] as important VOCs against *R. solani*. *T. harzianum* can produce also 3-octanone and 1-octen-3-ol, 2-phenylethyl alcohol and isopentyl acetate [29], compounds with fungistatic and fungicidal activity. Additionally, previous studies showed the production of 6-pentyl-2H-pyran-2-one (6PP) by several *Trichoderma* spp., including *T. harzianum*. 6PP is a secondary metabolite with inhibitory activity against several plant pathogenic fungi such as *R. solani* and *F. oxysporum* [60]. Nevertheless, it is impossible to pinpoint any specific compound without proper identification and individual testing, as hundreds of VOCs produced by *T. harzianum* and related species have been described so far [61,62].

A similar outcome was observed with *F. oxysporum* F3 using DDS and non-vented VOC chambers, with important PI when confronted to the T34 strain, and a reduction of this activity when using E20, albeit not statistically significant in some cases when using the DDS method. Nevertheless, both T34 and E20 strains demonstrated a completely opposite effect when they were tested in vented conditions. These vented treatments produced a significant growth promotion on *F. oxysporum* F3. Moreover, E20 showed an increase in this growth-promoting activity in comparison to the original T34 strain. In addition to the previous explanation, this could mean that the silencing of the *erg1* gene not only reduces the production of antifungal volatiles, but may also increase the production of growth-promoting bioactive compounds, at least in vented conditions. This could mean that squalene or some derived compound exerts this promoting activity in *F. oxysporum*. Alternatively, the explanation could be that the same compounds showing inhibitory activity in closed circumstances exert promoting effects when they act in vented ones. Notwithstanding, a combination of various complex and synergistic interactions could lay behind the reported effects, as stated by Strobel et al. [63]. VOCs may not be the only factor to explain the effects observed using these strains, as inorganic volatiles like CO_2_, NH_3_, or HCN could also be responsible for part of this basal activity in the wild type strain and, therefore, in its transformants. Possible changes in the pH of the growth medium due to the presence of inorganic volatiles could also account for some of the activity.

On the other hand, the results obtained from the T34-5.27 and E20-5.7 transformants revealed that the heterologous expression of *T. arundinaceum tri5* significantly increased the inhibitory effects of the overall VOCs produced by both strains. Interestingly, when using the DDS method, this effect was barely noticeable and not statistically significant in many cases. Indicating, probably, that the maximum inhibitory activity in tightly closed environments is reached without the need for this modification. On the contrary, assays using non-vented and vented VOC chambers showed the relevant effect of this modification, being not only able to increase the inhibitory capacity of the *Trichoderma* strains, but also reverting the effects observed using a vented VOC chamber, from the aforementioned significant growth promotion of T34 and E20 over *F. oxysporum* F3, to its significant inhibition. These findings support the results described by Malmierca et al. [42,43], where an increase in the antifungal activity of *Trichoderma* was also found using the same transformant strains against several fungal phytopathogens using confrontation and membrane assays but non-volatile confrontation assays. Besides, a recent study conducted by Taylor et al. [55] demonstrated that the overproduction of trichodiene by *T. harzianum* reduced the production of the mycotoxin deoxynivalenol by *F. graminearum*.

### 4.4. Additional Observations and Final Overview

Finally, VOC chambers were demonstrated to be easier and faster to use than the traditional DDS method. The time required to assemble the VOC chambers was much lower than the time needed to correctly place both Petri plates using the DDS method and seal them with parafilm. This is especially true for the vented chambers, while the non-vented prototypes used in this study still needed to be sealed with parafilm to ensure a fairly closed environment. Nevertheless, the time needed for the assembling of non-vented VOC chambers could be significantly reduced by its industrial production using an ad hoc locking system (e. g., Figure S2, numbers 10 and 11). Future assays could be performed with VOC chambers using membranes of filters to cover the central orifice, thus avoiding cross-contamination or even allowing for eventual compound sorting. These new features could be attached commercially or added in the laboratory. Moreover, the VOC chambers are not limited to use with filamentous fungi, nor even to microorganisms, but could be useful for evaluating VOC interactions involving other types of organisms. The VOC chamber could present new configurations and features with an outlet or adaptor to allow the direct extraction of the VOCs retained in the headspace, trapping them for ulterior analysis by GC/MS. Additionally, new future uses could arise from the scientific community for this novel technology.

In the end, the overall evidence from this study indicates that ventilation and gas exchange play a very important role in the microbial interactions mediated by VOCs. Furthermore, our findings highlight the limitations of the traditional DDS method on the performance of these experiments, demonstrating that VOC chambers have the ability to reveal differences that were not detected with the traditional method, showing higher selectivity between treatments in almost all of the tested circumstances, in addition to higher homogeneity of results. Tightly closed methods, such as the DDS, seem to be unable to reproduce the interactions developed between organisms in open environments. Therefore, we strongly believe that past and future VOC studies should be completed using the new techniques presented here. Thus, allowing for the assessment of the effect of different ventilation conditions on the microbial interactions mediated by volatile compounds. Nevertheless, we do not imply that previous studies using DDS or related methods are wrong, but rather that their findings and derived conclusions can be only applied to the airtight conditions tested, as already stated by some authors [32]. Therefore, the present work provides useful novel tools to solve these limitations, and opens up the opportunity for new trials to complete that past research, and to obtain a deeper understanding of microbial interactions. Consequentially, further and more specific investigations are strongly recommended for unveiling the very complex VOC-mediated microbial interactions that take place in different ecosystems and conditions, alongside their ecological, molecular, physiological, and genetic implications.

## 5. Patents

The device herein presented has been patented with the number ES 2708899 B2 in the Spanish Office of Patents and Trademarks and submitted with the international PCT number PCT/ES2019/070475. Thus far, additional patent applications have been already submitted to the following countries: United States of America, Canada, Japan, and the European Union. European Patent Office; Application Number 19846227.7 – 1132.

## Figures and Tables

**Table 1:** Coefficient of variation (CV) from *R. solani* R43 and *F. oxysporum* F3 growth using the DDS method, non-vented, and vented VOC chambers. In the table are represented the CV mean, its standard deviation (SD), and its standard error (SE). The results were analysed with one-way analysis of variance (ANOVA, *p* ≤ 0.05) and Tukey’s post hoc test (*p* ≤ 0.05). Different capital letters represent significant differences between treatments. The results from experiment 1 and experiment 2 are represented separately.

	Coefficient of Variation R43 Experiment 1			
Method	Mean	SD	SE	Statistics
DDS	0.156	0.083	0.019	A
Non-vented	0.048	0.024	0.005	B
Vented	0.057	0.040	0.009	B
	**Coefficient of Variation R43 Experiment 2**			
Method	Mean	SD	SE	Statistics
DDS	0.10	0.056	0.012	A
Non-vented	0.040	0.016	0.004	B
Vented	0.069	0.039	0.009	B
	**Coefficient of Variation F3 Experiment 1**			
Method	Mean	SD	SE	Statistics
DDS	0.062	0.037	0.010	A
Non-vented	0.035	0.027	0.007	B
Vented	0.026	0.014	0.004	B
	**Coefficient of Variation F3 Experiment 2**			
Method	Mean	SD	SE	Statistics
DDS	0.050	0.015	0.004	A
Non-vented	0.021	0.008	0.002	B
Vented	0.031	0.015	0.004	B

## Data Availability

The data that support the findings of this study are available in Supplementary materials and additional data can be obtained from the corresponding author upon reasonable request. All microbiological strains used in this study will be made available to researchers upon reasonable request. After publication, VOC chambers will be made available to researchers upon reasonable request, unless commercial agreements reached with third parties regarding the patent exploitation prohibit it (in which case the VOC chambers should be available in the market). Correspondence and requests for materials should be addressed to S.A.-G.

## Supplementary Materials

The following are available online at https://www.mdpi.com/2309-608X/7/4/248/s1, Supplementary Figure S1, Supplementary Figure S2, Supplementary Figure S3, Plan 1A, Plan 2A, Plan 3A, Plan 1B, Plan 2B, Supplementary Table S1, Supplementary Table S2, Supplementary Table S3, and Supplementary Table S4.
