# Supplementary material for: Volatile Organic Compound Chamber: A Novel Technology for Microbiological Volatile Interaction Assays
Source: J Fungi (Basel). 2021 Mar 25;7(4):248. doi: 10.3390/jof7040248 (PMC8064445; doi:10.3390/jof7040248)
Supplement: Supplementary file 1 [file jof-07-00248-s001.pdf]

SUPPLEMENTARY MATERIALS

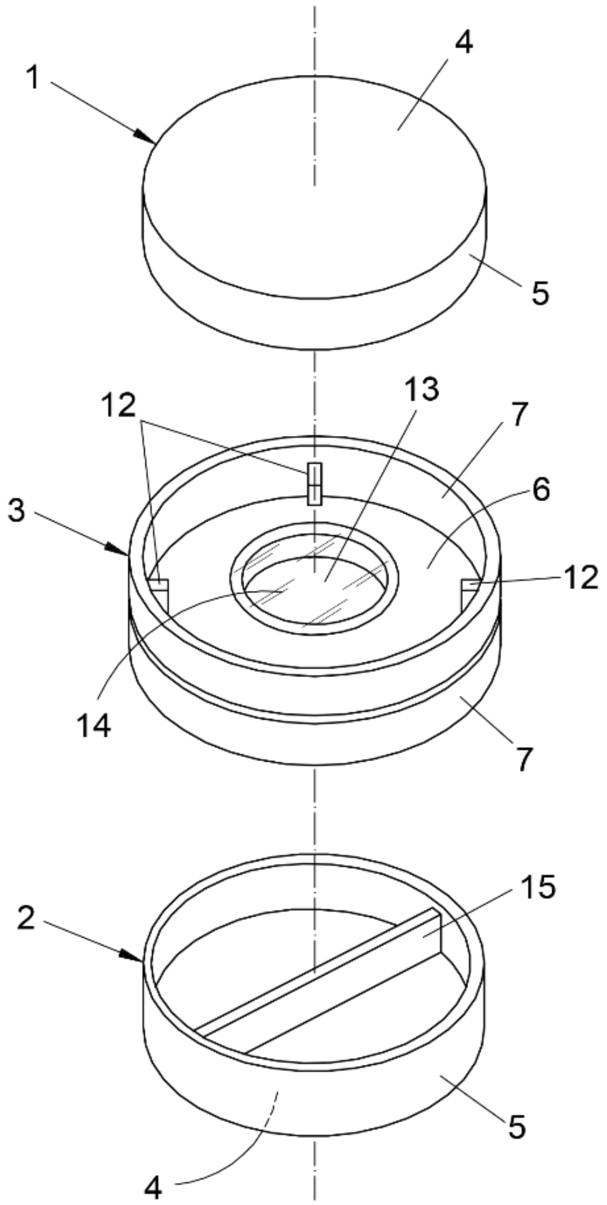

Supplementary Figure SF1. Explosive View. Vented VOC Chamber. 1: upper plate; 2: lower plate; 3: central piece; 4: upper and lower walls (plates); 5: perimeter wall (plates); 6: intermediate wall (central piece); 7: lateral walls (central piece); 8: upper face (not shown. See SF2); 9: lower face (not shown. See SF2); 10: internal edge (not shown. See SF2); 11: shelter (not shown. See SF2); 12: ventilation flanges; 13: central hole; 14: membrane or filter (optional. Not used in this study); 15: septum for divided Petri dishes (optional. Not used in this study).

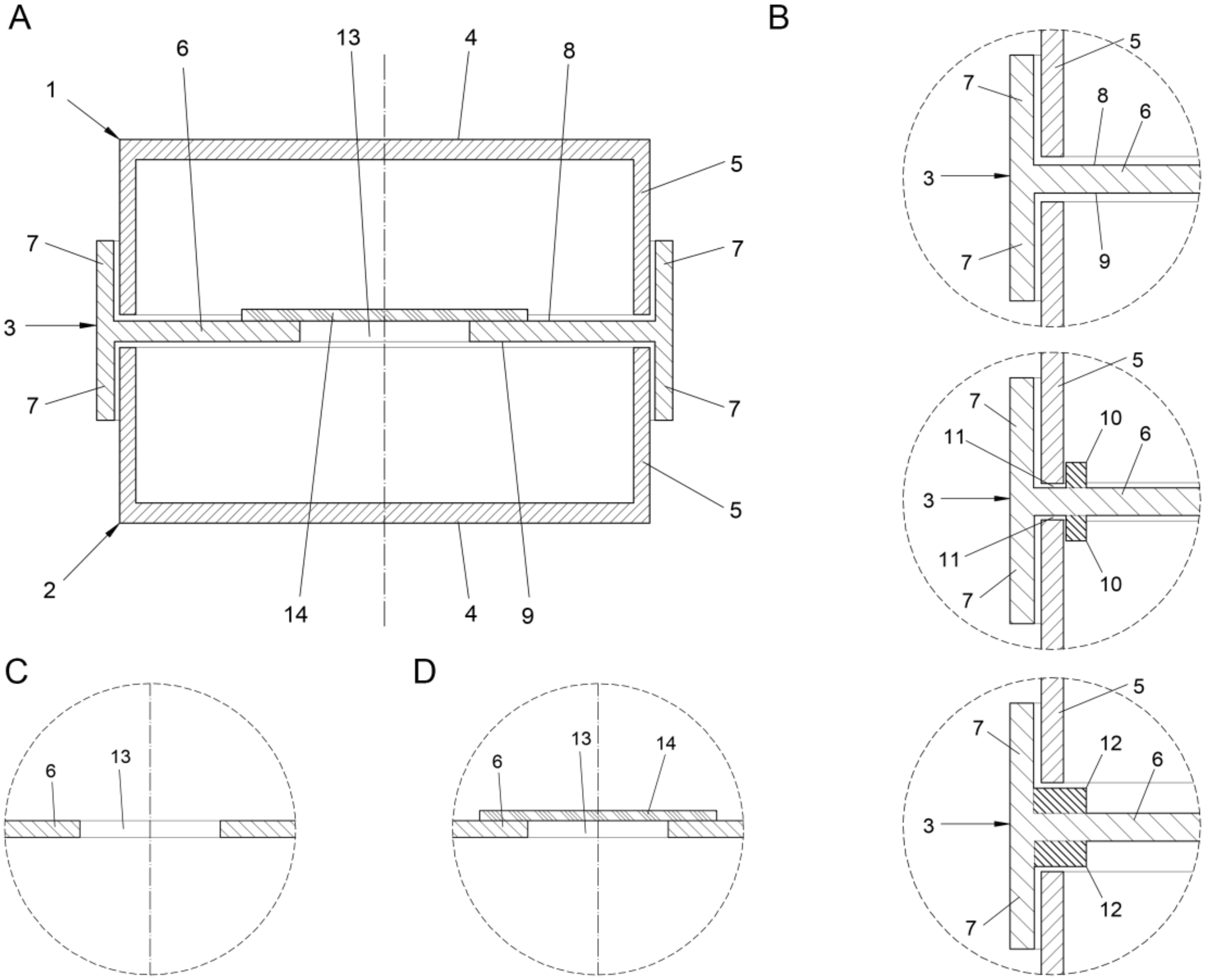

Supplementary Figure SF2. (A) Frontal Cross-section. Non-Vented VOC Chamber. **(B)** Detail of union between plates and central piece in Non-Vented VOC Chambers (upper circle), Non-Vented VOC Chamber with locking system (central circle), Vented VOC Chamber with flanges (lower circle). **(C)** Detail of central hole without membrane or filter. **(D)** Detail of central hole with membrane or filter attached. 1: upper plate; 2: lower plate; 3: central piece; 4: upper and lower walls (plates); 5: perimeter wall (plates); 6: intermediate wall (central piece); 7: lateral walls (central piece); 8: upper face (central piece); 9: lower face (central piece); 10: internal edge (locking system. Optional. Not used in this study); 11: shelter (locking system. Optional. Not used in this study); 12: ventilation flanges; 13: central hole; 14: membrane or filter (optional. Not used in this study).

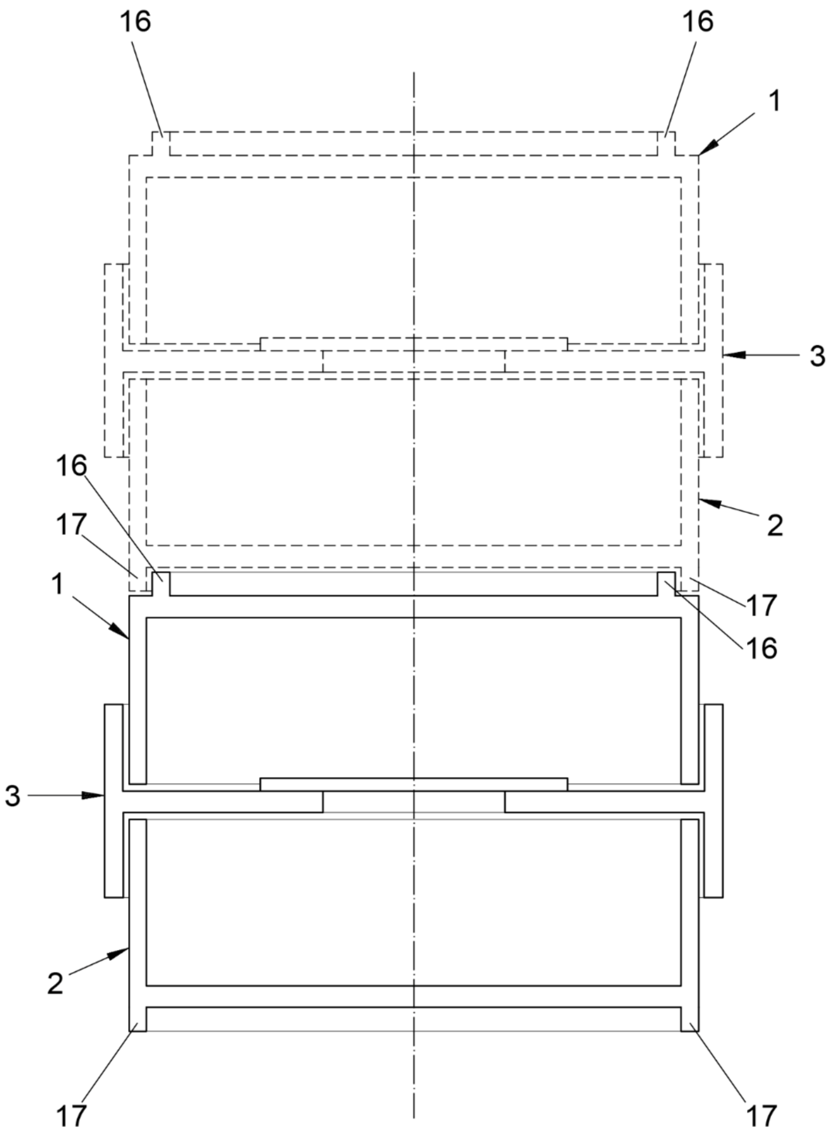

Supplementary Figure SF3. Frontal Cross-section of two Non-Vented VOC Chambers stacked. 1: upper plate; 2: lower plate; 3: central piece; 16: upper inner ring for stacking chambers (optional. Not used in this study); 17: lower outer ring for stacking chambers (optional. Not used in this study).

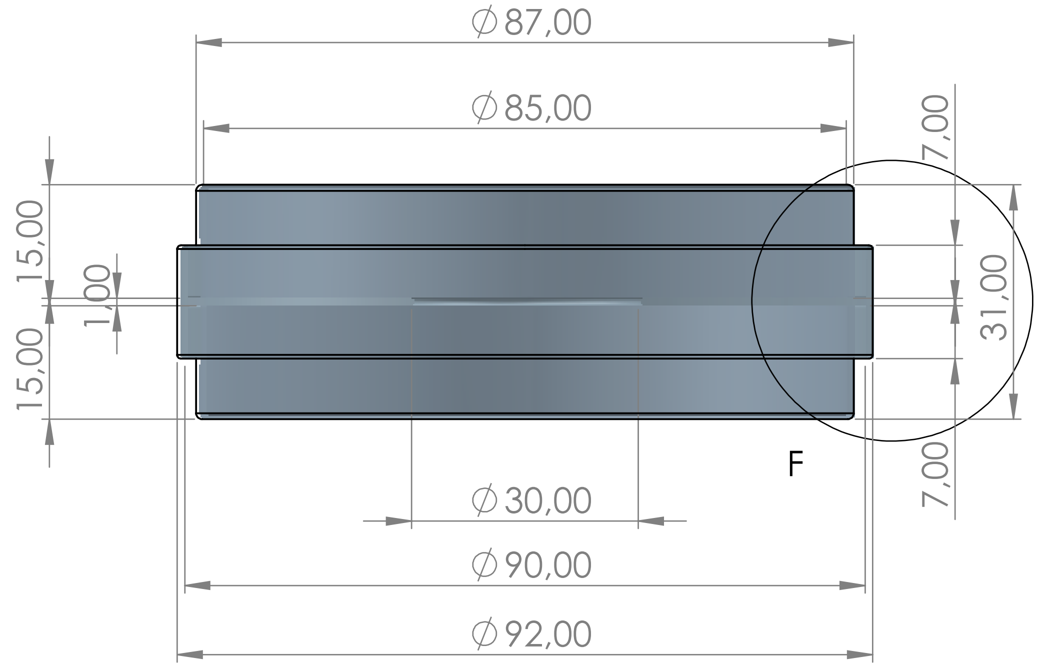

FRONT VIEW

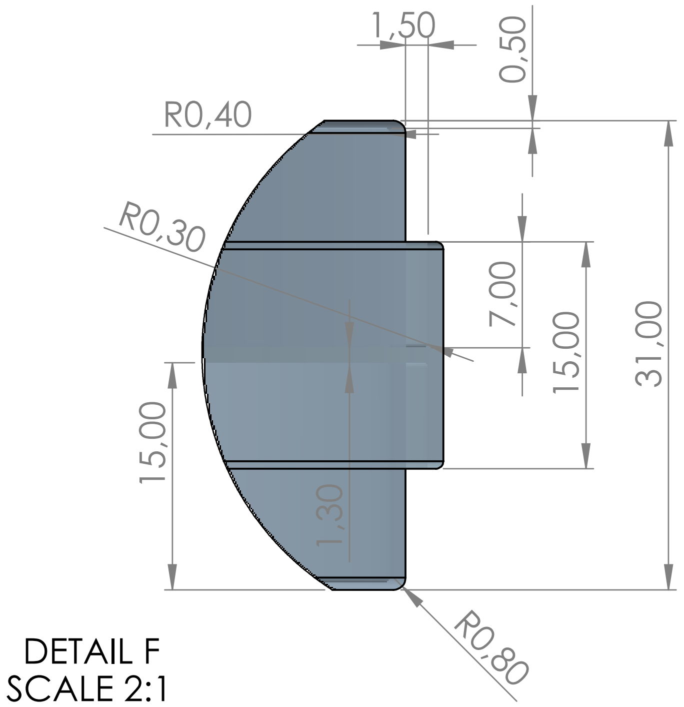

DETAIL F
SCALE 2:1

DETAIL UNION BETWEEN CENTRAL PIECE AND PLATE

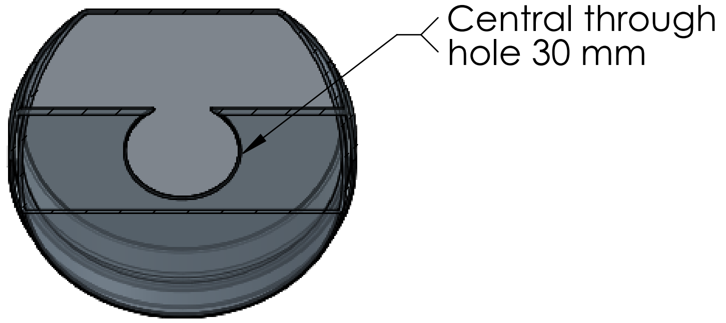

SECTION B-B
SCALE 1 : 2

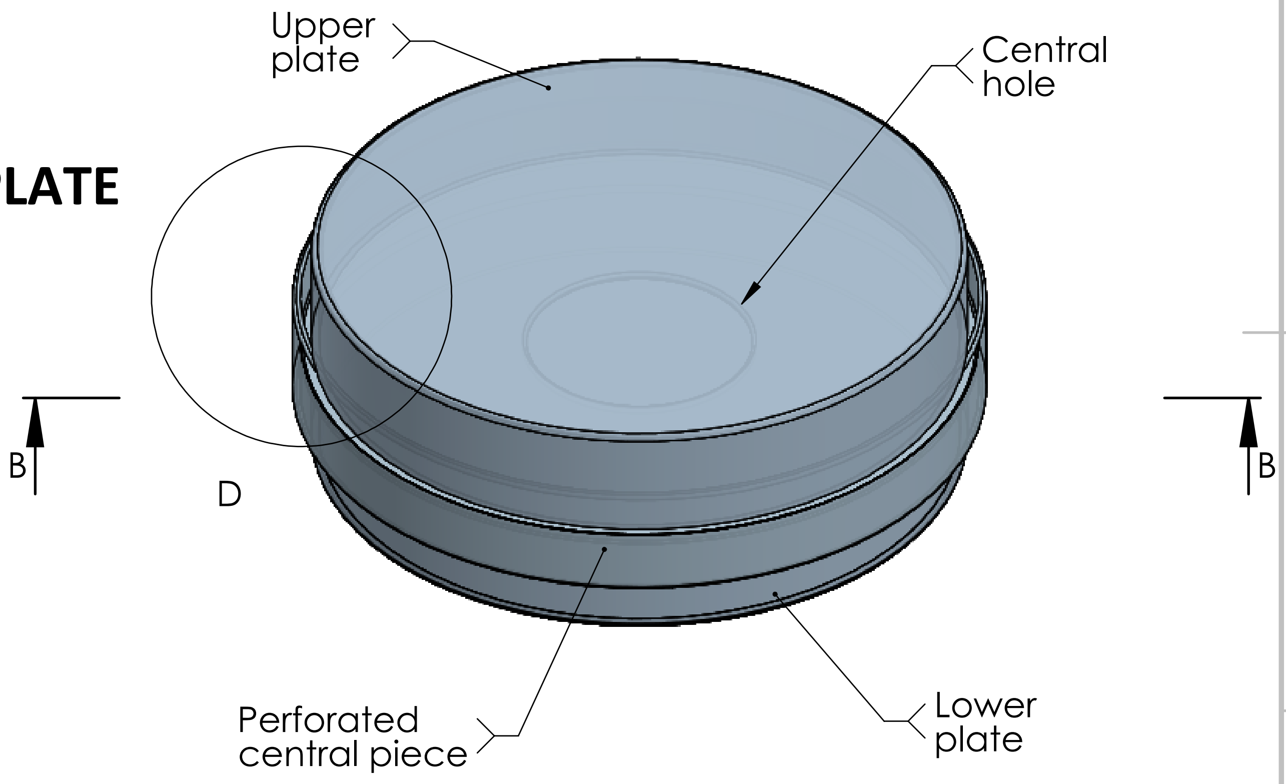

AUXILIARY VIEW

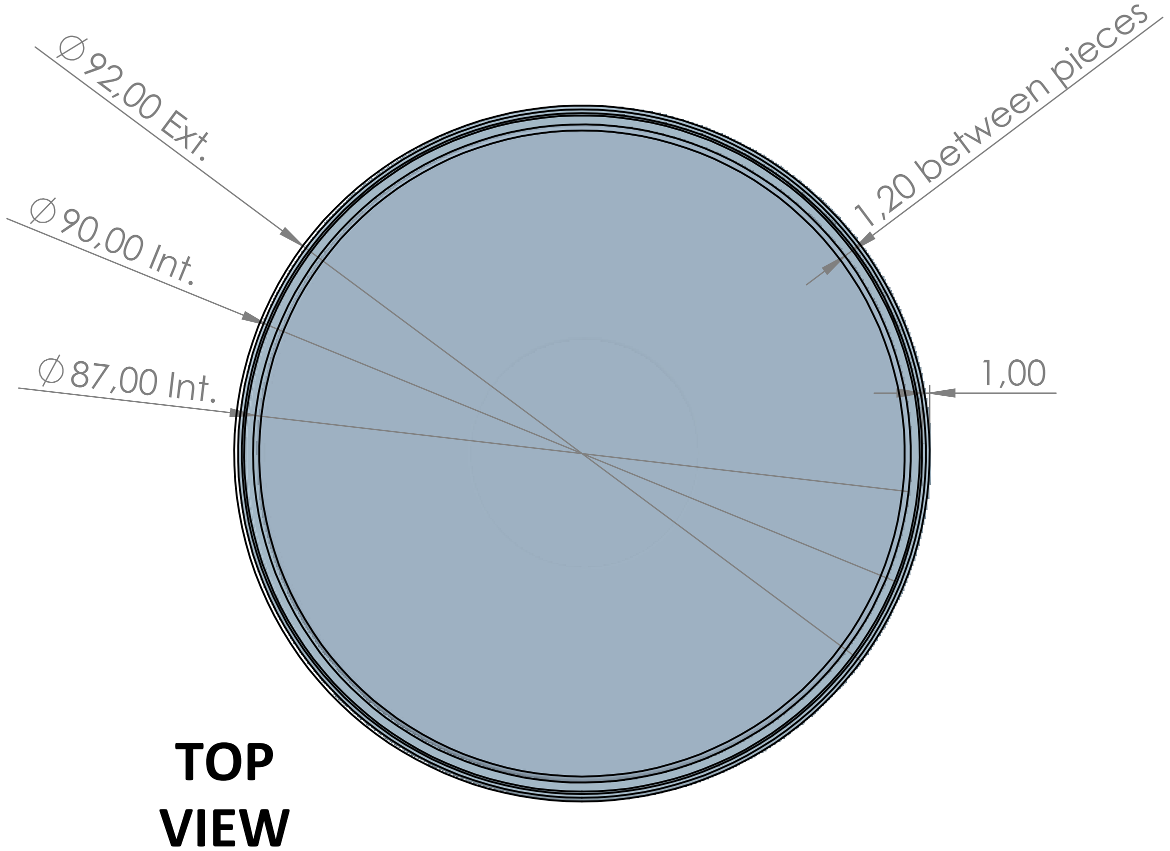

TOP VIEW

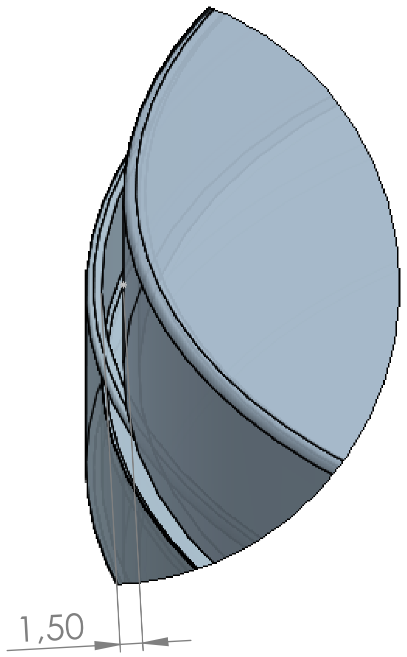

DETAIL D
SCALE 2:1

**UNIVERSITY OF LEÓN**			
VOLATILE ORGANIC COMPOUNDS CHAMBER - VOC CHAMBER			
PLAN:	Full Non - Vented VOC Chamber		
SCALA: 1 : 1	Plan drawn by:	Mario del Val Martínez	Plan: **1A**
DATE: April 2020			

1 x Central piece per full chamber

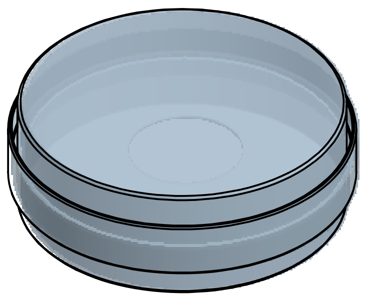

Scale: 1/2

FULL CHAMBER

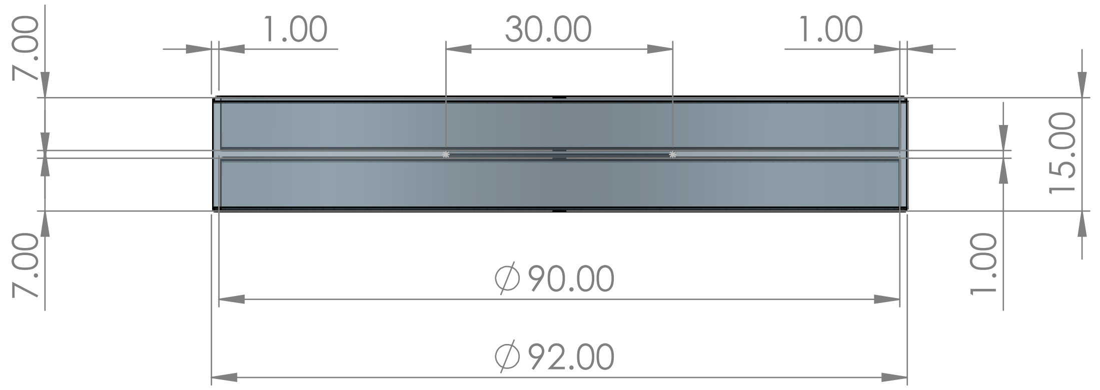

FRONT VIEW

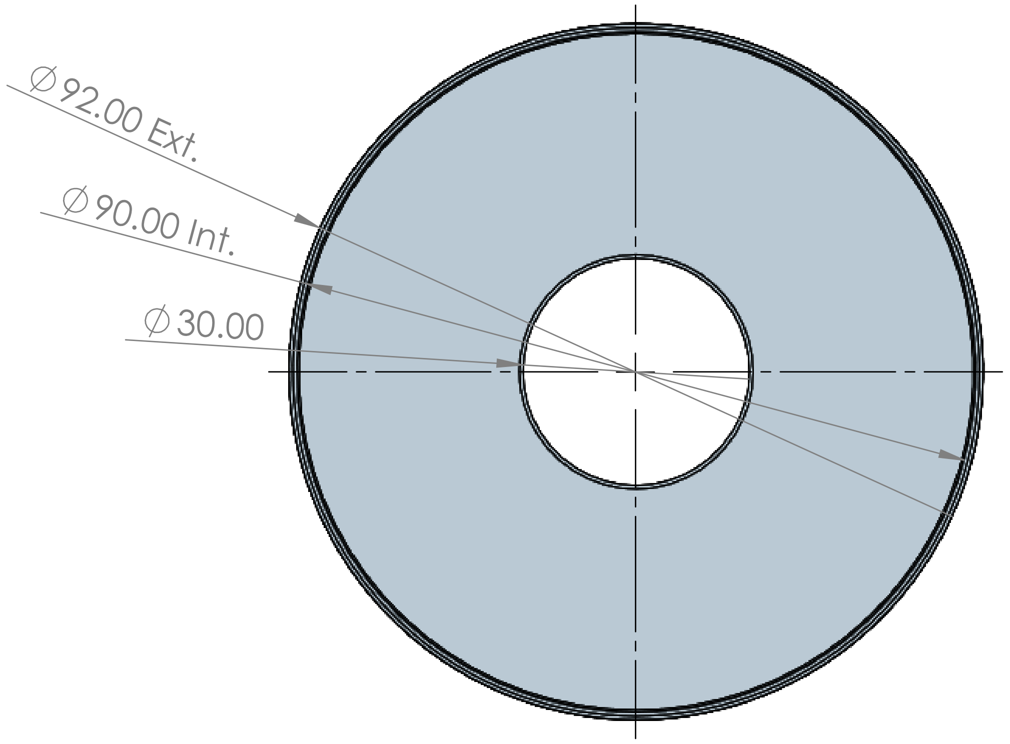

TOP VIEW

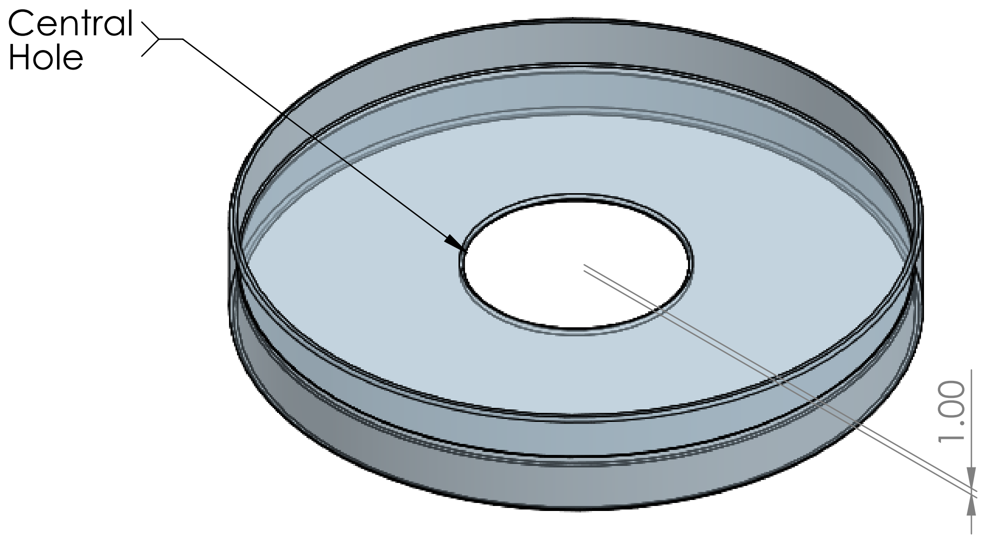

AUXILIARY VIEW

**UNIVERSITY OF LEÓN**		
VOLATILE ORGANIC COMPOUNDS CHAMBER<br>VOC CHAMBER		
PLAN: Perforated central piece		
SCALE: 1 : 1	Plan drawn by: Mario del Val Martínez	Plan: **2A**
DATE: April 2020		

2 x Petri plates per full chamber

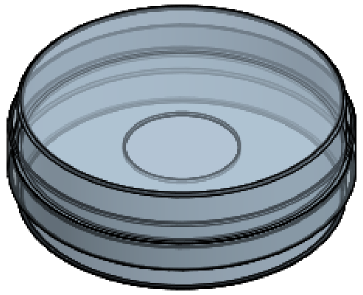

Scale: 1/2

FULL CHAMBER

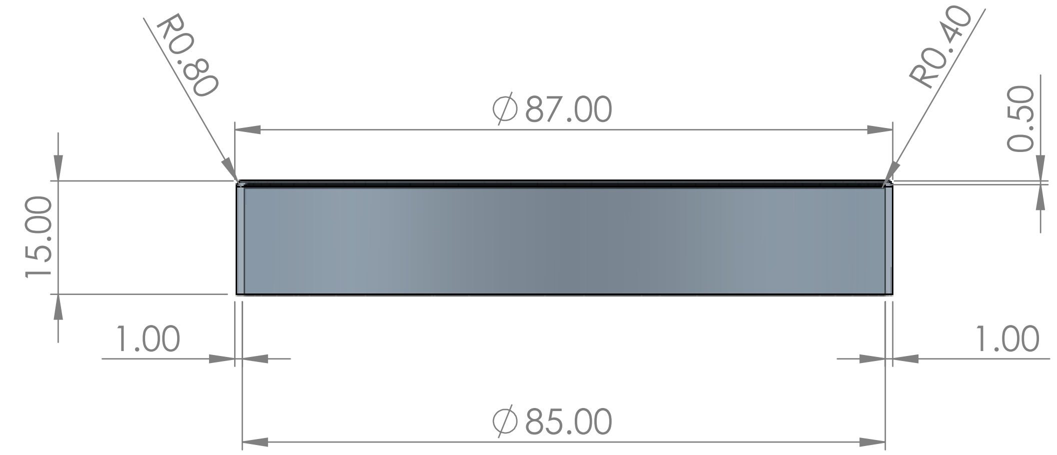

FRONT VIEW

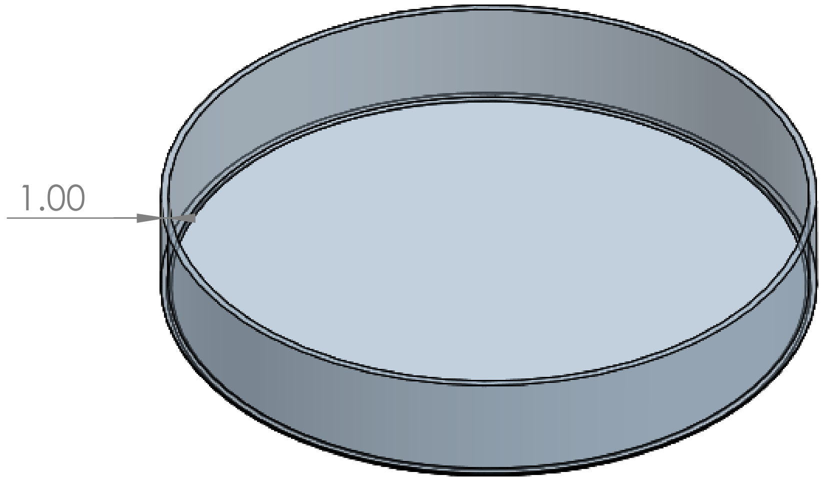

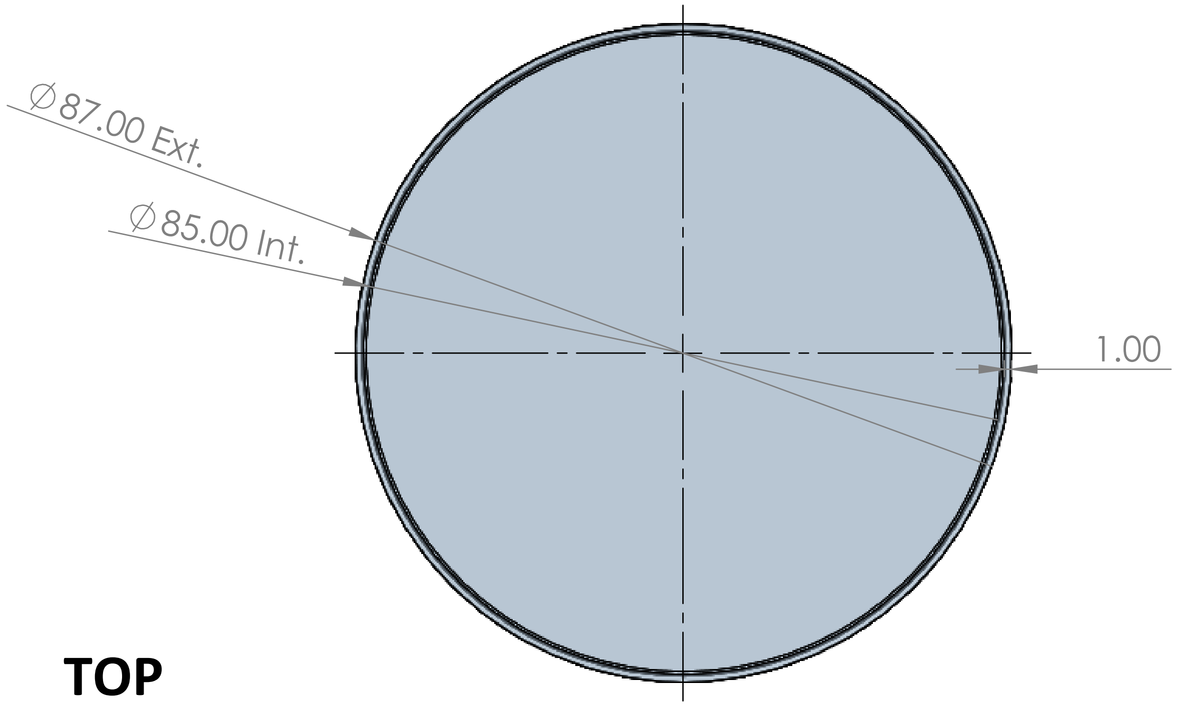

AUXILIARY VIEW

TOP VIEW

UNIVERSITY OF LEÓN			
VOLATILE ORGANIC COMPOUNDS CHAMBER<br>VOC CHAMBER			
PLAN:	Plates		
SCALE:	1 : 1	Plan drawn by:<br>Mario del Val Martínez	Plan:<br>**3A**
DATE:	April 2020		

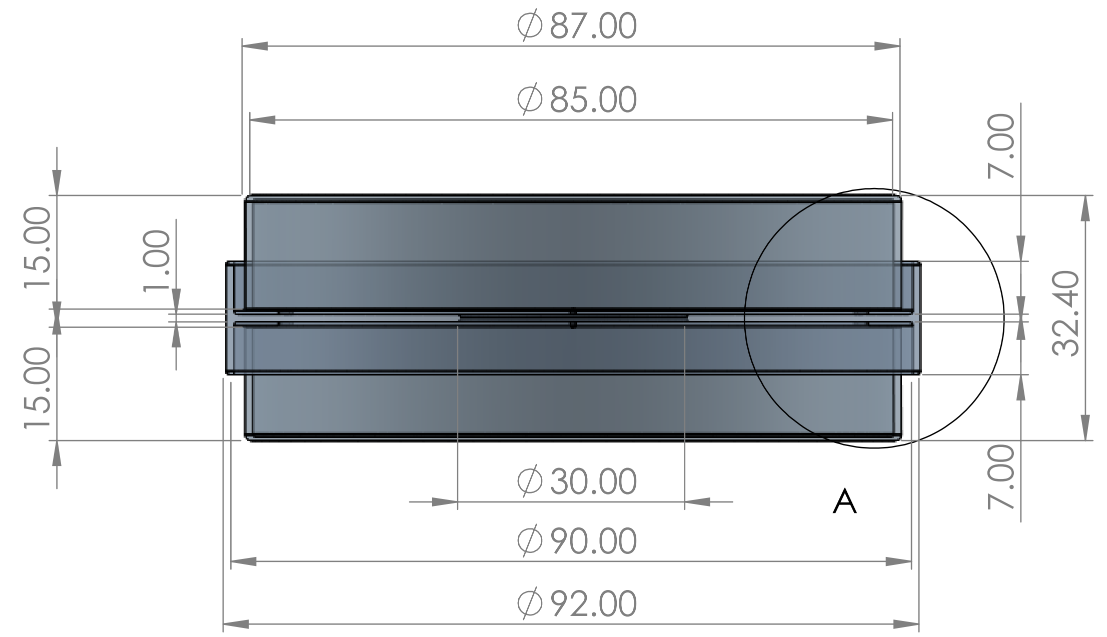

FRONT VIEW

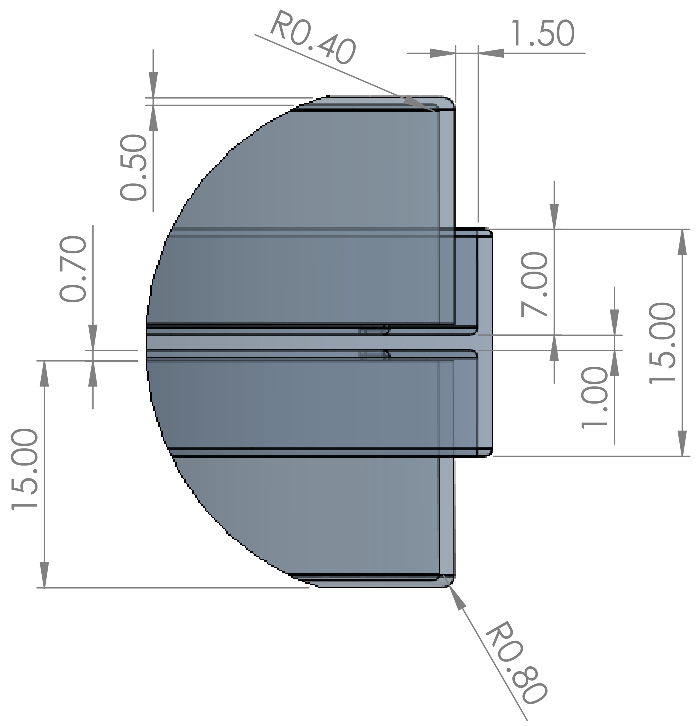

DETAIL A
SCALE 2:1

AUXILIARY VIEW

DETAIL UNION BETWEEN CENTRAL PIECE AND PLATE

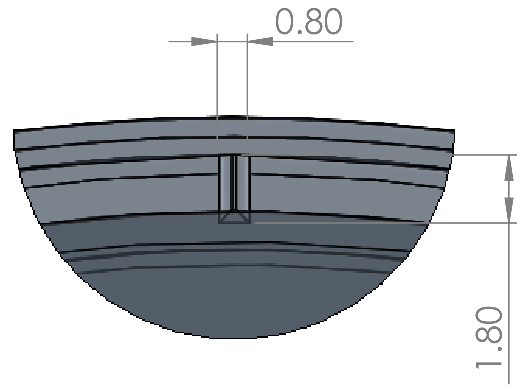

DETAIL C
SCALE 5:1

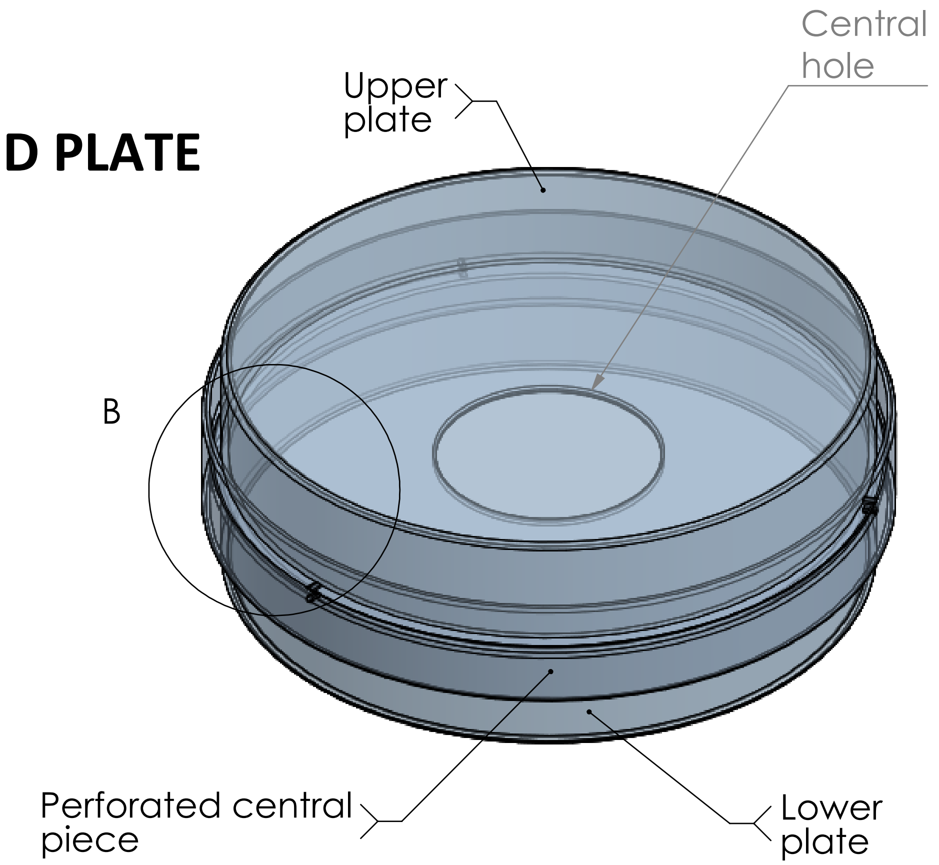

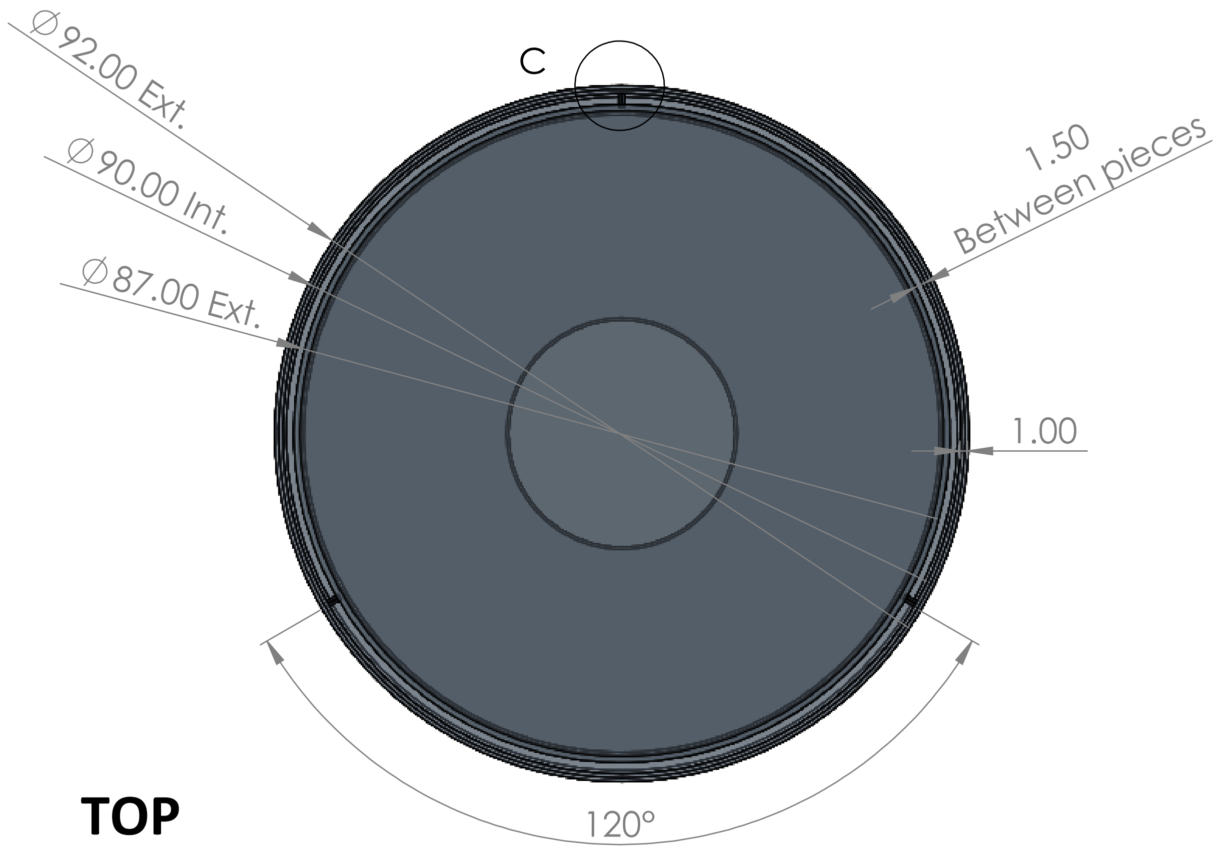

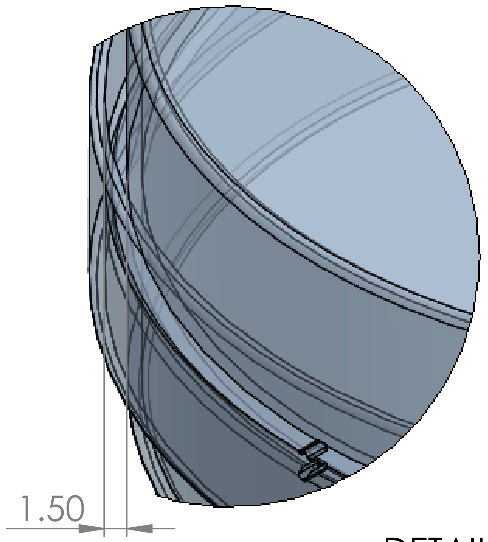

DETAIL B
SCALE 2:1

TOP VIEW

UNIVERSITY OF LEÓN			
VOLATILE ORGANIC COMPOUNDS CHAMBER<br>VOC CHAMBER			
PLAN:	Full Vented VOC Chamber		
SCALA: 1 : 1	Plan drawn by: Mario del Val Martínez		Plan: **1B**
DATE: April 2020			

1 x Central piece per full chamber

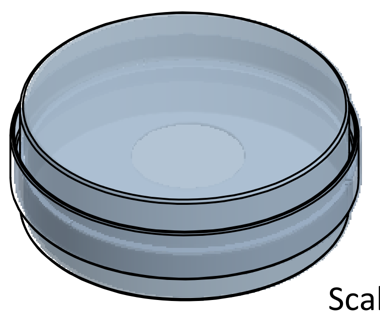

FULL CHAMBER

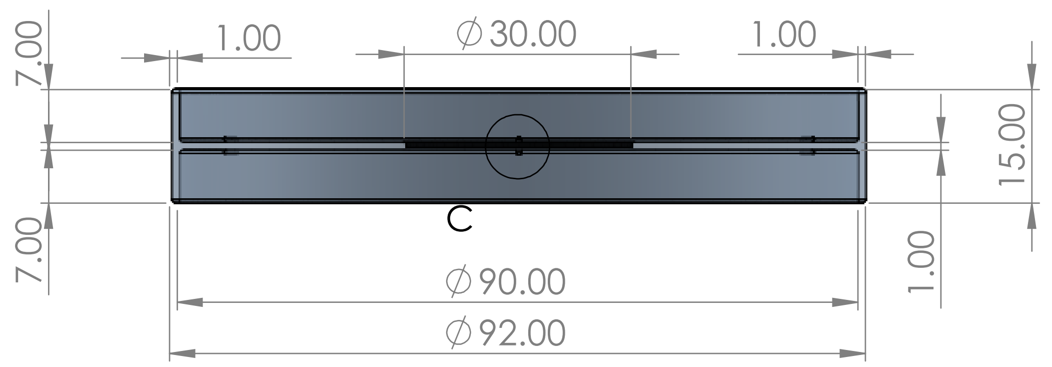

FRONT VIEW

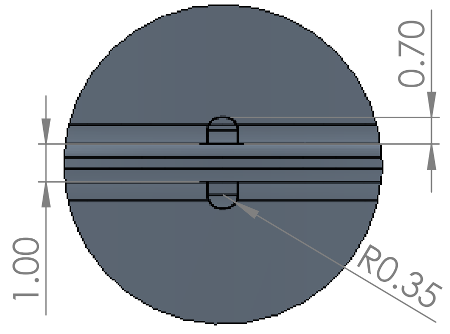

DETAIL C
SCALE 5:1

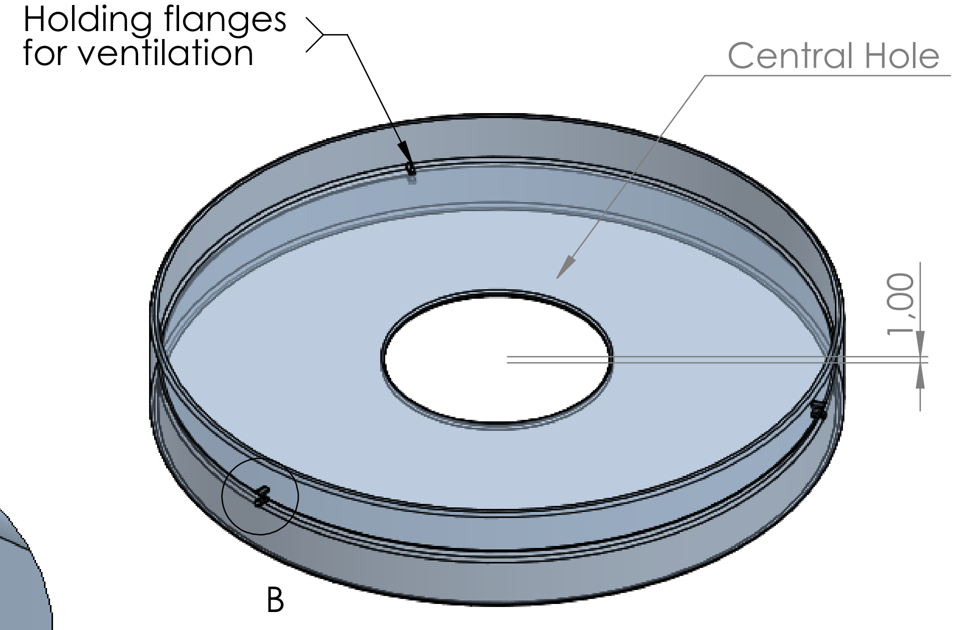

AUXILIARY VIEW

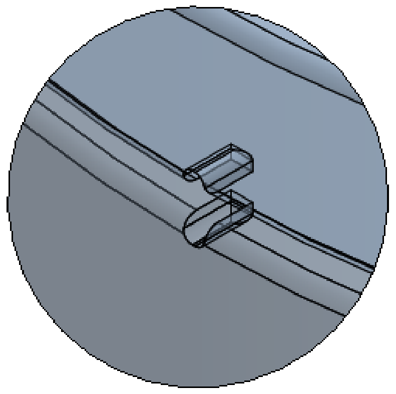

DETAIL B
SCALE 5:1

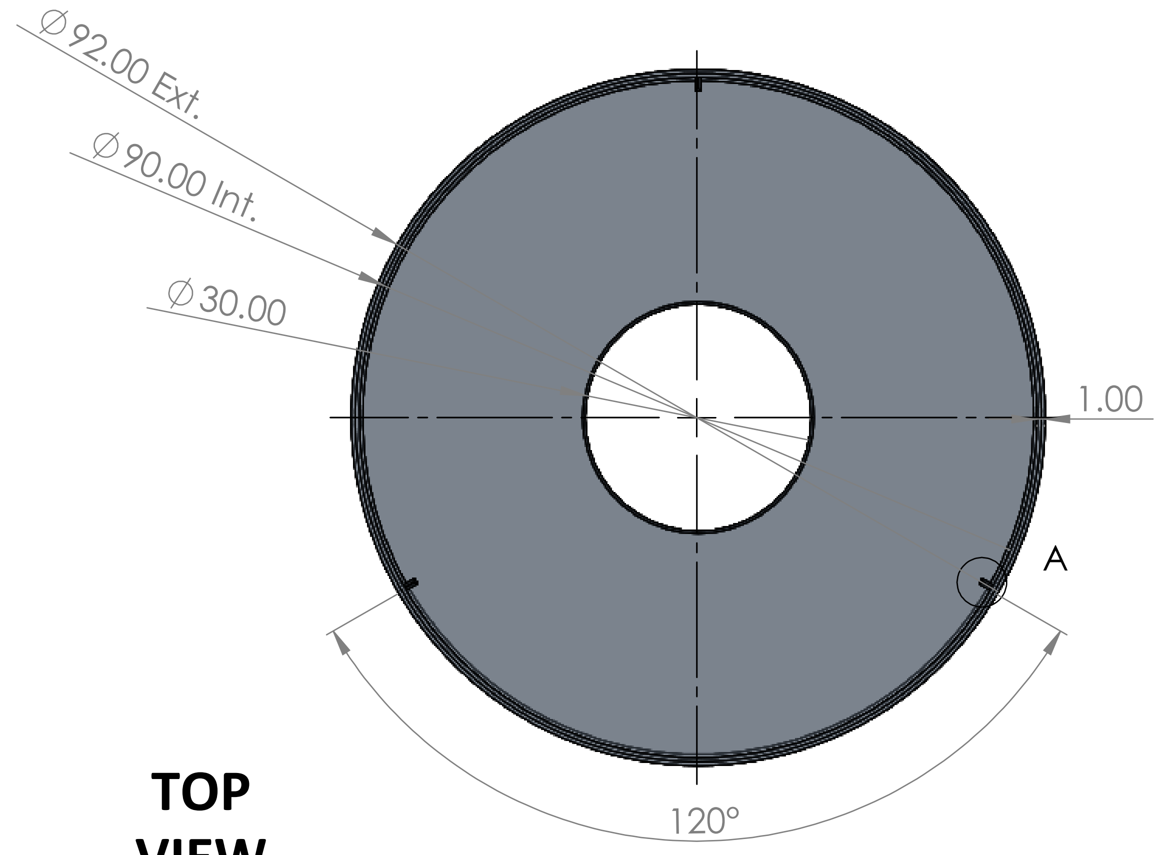

TOP VIEW

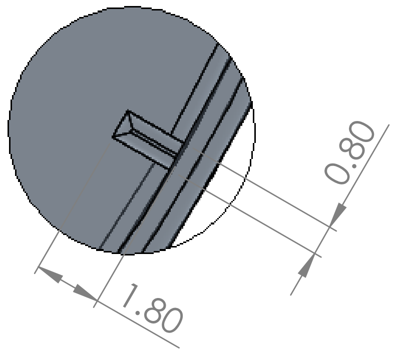

DETAIL A
SCALE 5 :1

**UNIVERSITY OF LEÓN**			
VOLATILE ORGANIC COMPOUNDS CHAMBER<br>VOC CHAMBER			
PLAN:	Perforated central piece		
SCALA: 1 : 1	Plan drawn by:	Mario del Val Martínez	Plan: **2B**
DATE: April 2020			

Supplementary table ST1. Experiment 1. Growth (mm) and Percentage of inhibition (PI) of *R. solani* R43 confronted by *T. harzianum* T34, T34-5.27, E20 and E20-5.7 using DDS method (first row), Non-Vented VOC Chambers (second row), and Vented VOC Chambers (third row); after 1, 2, 3, and 4 days post-inoculation. PI was calculated in relation to the control using the following equation proposed by Gotor-Vila et al. (2017): [(C-T) / C] x 100. Where C is the diameter of the controls and T that of the treatments. PI is expressed as the mean of the four replicates. P values were obtained from growth results using one-way analysis of variance (ANOVA), after confirmation of normality and equality of variances, and were contrasted using Tukey's *post hoc* test. Capital letters represent the differences between treatments with P ≤ 0.05; P ≤ 0.01; and P ≤ 0.001, respectively. All statistical analyses were performed using SPSS 24.0 (IBM).

R43 EXPERIMENT 1		day 1						day 2						day 3						day 4					
		GROWTH (mm)		INHIBITION (%)	STATISTICS			GROWTH (mm)		INHIBITION (%)	STATISTICS			GROWTH (mm)		INHIBITION (%)	STATISTICS			GROWTH (mm)		INHIBITION (%)	STATISTICS		
	TREATMENT	Mean	SD	Mean	P ≤ 0.05	P ≤ 0.01	P ≤ 0.001	Mean	SD	Mean	P ≤ 0.05	P ≤ 0.01	P ≤ 0.001	Mean	SD	Mean	P ≤ 0.05	P ≤ 0.01	P ≤ 0.001	Mean	SD	Mean	P ≤ 0.05	P ≤ 0.01	P ≤ 0.001
**Double Dish Set**	CC	17.92	1.23	-	A	A	A	34.9167	4.11	-	A	A	A	45.50	3.19	-	A	A	A	54.58	2.63	-	A	A	A
	R43/T34	10.00	1.05	44.19	C	BC	BC	13.25	1.91	62.05	BC	B	B	15.00	2.94	67.03	B	B	B	16.00	3.47	70.69	B	B	B
	R43/T34-5.27	6.92	0.17	61.40	D	D	C	9.16667	1.35	73.75	D	B	B	10.58	2.04	76.74	B	B	B	12.25	3.41	77.56	B	B	B
	R43/E20	12.25	1.29	31.63	B	B	B	15.9167	3.98	54.42	B	B	B	17.58	5.03	61.36	B	B	B	18.42	5.82	66.26	B	B	B
	R43/E20-5.7	7.50	0.58	58.14	D	CD	C	11.5833	1.69	66.83	BC	B	B	14.75	2.32	67.58	B	B	B	17.08	2.91	68.70	B	B	B

		GROWTH (mm)		INHIBITION (%)	STATISTICS			GROWTH (mm)		INHIBITION (%)	STATISTICS			GROWTH (mm)		INHIBITION (%)	STATISTICS			GROWTH (mm)		INHIBITION (%)	STATISTICS		
	TREATMENT	Mean	SD	Mean	P ≤ 0.05	P ≤ 0.01	P ≤ 0.001	Mean	SD	Mean	P ≤ 0.05	P ≤ 0.01	P ≤ 0.001	Mean	SD	Mean	P ≤ 0.05	P ≤ 0.01	P ≤ 0.001	Mean	SD	Mean	P ≤ 0.05	P ≤ 0.01	P ≤ 0.001
**Non-Vented VOC Chaber**	CC	19.75	0.74	-	A	A	A	36.67	1.12	-	A	A	A	51.25	1.50	-	A	A	A	63.58	1.71	-	A	A	A
	R43/T34	13.42	0.32	32.07	C	B	B	19.75	0.32	46.14	C	C	BC	24.50	1.40	52.20	C	C	BC	33.00	3.32	48.10	BC	BC	BC
	R43/T34-5.27	8.08	0.32	59.07	D	C	C	13.75	1.03	62.50	D	D	D	19.67	1.31	61.63	D	D	C	28.00	2.19	55.96	CD	C	BC
	R43/E20	15.08	0.83	23.63	B	B	B	23.42	0.63	36.14	B	B	B	29.25	0.63	42.93	B	B	B	36.50	1.11	42.60	B	B	B
	R43/E20-5.7	9.08	0.63	54.01	D	C	C	15.83	0.58	56.82	D	D	CD	20.33	1.45	60.33	D	D	C	27.56	1.68	56.66	D	C	C

		GROWTH (mm)		INHIBITION (%)	STATISTICS			GROWTH (mm)		INHIBITION (%)	STATISTICS			GROWTH (mm)		INHIBITION (%)	STATISTICS			GROWTH (mm)		INHIBITION (%)	STATISTICS		
	TREATMENT	Mean	SD	Mean	P ≤ 0.05	P ≤ 0.01	P ≤ 0.001	Mean	SD	Mean	P ≤ 0.05	P ≤ 0.01	P ≤ 0.001	Mean	SD	Mean	P ≤ 0.05	P ≤ 0.01	P ≤ 0.001	Mean	SD	Mean	P ≤ 0.05	P ≤ 0.01	P ≤ 0.001
**Vented VOC Chaber**	CC	19.08	1.00	-	A	A	A	40.83	0.79	-	A	A	A	60.83	1.69	-	A	A	A	71.75	1.07	-	A	A	A
	R43/T34	19.00	0.98	0.44	A	A	A	38.50	3.49	5.71	A	A	A	53.08	7.05	12.74	A	A	A	63.08	2.57	12.08	B	B	B
	R43/T34-5.27	10.00	0.27	47.60	B	B	B	19.25	0.32	52.86	B	B	B	26.67	0.47	56.16	B	B	B	32.50	0.58	54.70	C	C	C
	R43/E20	19.67	1.31	-3.06	A	A	A	41.11	4.74	4.90	A	A	A	51.89	7.82	14.70	A	A	A	65.11	2.59	9.25	B	B	AB
	R43/E20-5.7	11.75	0.50	38.43	B	B	B	21.75	1.40	46.73	B	B	B	29.58	2.47	51.37	B	B	B	34.75	2.13	51.57	C	C	C

Supplementary table ST2. Experiment 2. Growth (mm) and Percentage of inhibition (PI) of *R. solani* R43 confronted by *T. harzianum* T34, T34-5.27, E20, and E20-5.7 using DDS method (first row), Non-Vented VOC Chambers (second row), and Vented VOC Chambers (third row); after 1, 2, 3, and 4 days post-inoculation. PI was calculated in relation to the control using the following equation proposed by Gotor-Vila et al. (2017): [(C-T) / C] x 100. Where C is the diameter of the controls and T that of the treatments. PI is expressed as the mean of the four replicates. P values were obtained from growth results using one-way analysis of variance (ANOVA), after confirmation of normality and equality of variances, and were contrasted using Tukey's *post hoc* test. Capital letters represent the differences between treatments with P ≤ 0.05; P ≤ 0.01; and P ≤ 0.001, respectively. All statistical analyses were performed using SPSS 24.0 (IBM).

R43 EXPERIMENT 2		day 1						day 2						day 3						day 4					
		GROWTH (mm)		INHIBITION (%)	STATISTICS			GROWTH (mm)		INHIBITION (%)	STATISTICS			GROWTH (mm)		INHIBITION (%)	STATISTICS			GROWTH (mm)		INHIBITION (%)	STATISTICS		
	TREATMENT	Mean	SD	Mean	P ≤ 0.05	P ≤ 0.01	P ≤ 0.001	Mean	SD	Mean	P ≤ 0.05	P ≤ 0.01	P ≤ 0.001	Mean	SD	Mean	P ≤ 0.05	P ≤ 0.01	P ≤ 0.001	Mean	SD	Mean	P ≤ 0.05	P ≤ 0.01	P ≤ 0.001
Double Dish Set	CC	15.33	0.47	-	A	A	A	31.42	1.03	-	A	A	A	49.25	2.63	-	A	A	A	62.92	3.30	-	A	A	A
	R43/T34	10.67	0.72	30.43	B	B	BC	15.17	1.11	51.72	C	C	BC	17.00	2.09	65.48	C	BC	BC	18.58	2.46	70.46	C	BC	BC
	R43/T34-5.27	7.00	0.47	54.35	C	C	D	10.58	1.29	66.31	D	D	D	12.58	2.51	74.45	C	C	C	13.58	3.38	78.41	C	C	C
	R43/E20	11.50	1.11	25.00	B	B	B	18.92	1.26	39.79	B	B	B	22.83	2.76	53.64	B	B	B	27.17	3.25	56.82	B	B	B
	R43/E20-5.7	8.50	0.43	44.57	C	C	CD	13.33	0.98	57.56	C	CD	CD	16.67	1.76	66.16	C	BC	BC	19.42	3.01	69.14	C	BC	BC

		GROWTH (mm)		INHIBITION (%)	STATISTICS			GROWTH (mm)		INHIBITION (%)	STATISTICS			GROWTH (mm)		INHIBITION (%)	STATISTICS			GROWTH (mm)		INHIBITION (%)	STATISTICS		
	TREATMENT	Mean	SD	Mean	P ≤ 0.05	P ≤ 0.01	P ≤ 0.001	Mean	SD	Mean	P ≤ 0.05	P ≤ 0.01	P ≤ 0.001	Mean	SD	Mean	P ≤ 0.05	P ≤ 0.01	P ≤ 0.001	Mean	SD	Mean	P ≤ 0.05	P ≤ 0.01	P ≤ 0.001
Non-Vented VOC Chaber	CC	15.67	0.72	-	A	A	A	38.42	1.13	-	A	A	A	50.25	2.41	-	A	A	A	65.00	3.80	-	A	A	A
	R43/T34	11.92	0.32	23.94	C	C	B	19.83	0.64	48.37	C	C	C	26.67	1.36	46.93	C	C	BC	34.25	3.01	47.31	BC	BC	B
	R43/T34-5.27	7.50	0.33	52.13	E	E	D	13.25	0.32	65.51	E	E	E	19.42	0.57	61.36	D	D	D	25.50	0.88	60.77	D	D	C
	R43/E20	13.33	0.47	14.89	B	B	B	23.83	0.58	37.96	B	B	B	31.08	0.74	38.14	B	B	B	38.08	0.96	41.41	B	B	B
	R43/E20-5.7	9.50	0.33	39.36	D	D	C	16.67	0.98	56.62	D	D	D	23.67	1.25	52.90	C	C	CD	30.42	1.29	53.21	CD	CD	BC

		GROWTH (mm)		INHIBITION (%)	STATISTICS			GROWTH (mm)		INHIBITION (%)	STATISTICS			GROWTH (mm)		INHIBITION (%)	STATISTICS			GROWTH (mm)		INHIBITION (%)	STATISTICS		
	TREATMENT	Mean	SD	Mean	P ≤ 0.05	P ≤ 0.01	P ≤ 0.001	Mean	SD	Mean	P ≤ 0.05	P ≤ 0.01	P ≤ 0.001	Mean	SD	Mean	P ≤ 0.05	P ≤ 0.01	P ≤ 0.001	Mean	SD	Mean	P ≤ 0.05	P ≤ 0.01	P ≤ 0.001
Vented VOC Chaber	CC	14.75	1.40	-	A	A	A	31.92	1.42	-	A	A	AB	51.26	2.33	-	A	A	A	60.67	0.98	-	A	A	A
	R43/T34	14.33	1.28	2.82	A	A	A	29.67	1.61	7.05	A	A	AB	48.67	4.21	4.58	A	A	A	53.67	4.15	11.54	AB	AB	AB
	R43/T34-5.27	9.33	0.27	36.72	B	B	B	16.58	1.87	48.04	C	C	C	23.58	3.66	53.76	C	C	B	29.58	3.07	51.24	C	C	C
	R43/E20	15.42	0.42	-4.52	A	A	A	30.92	2.47	3.13	A	A	A	46.83	5.27	8.17	A	A	A	56.58	6.29	6.73	A	A	AB
	R43/E20-5.7	10.58	0.50	28.25	B	B	B	23.22	0.51	32.64	B	B	BC	35.22	1.02	30.94	B	B	AB	45.33	1.45	25.27	B	B	B

Supplementary table ST3. Experiment 1. Growth (mm) and Percentage of inhibition (PI) of *F. oxysporum* F3 confronted by *T. harzianum* T34, T34-5.27, E20, and E20-5.7 using DDS method (first row), Non-Vented VOC Chambers (second row), and Vented VOC Chambers (third row); after 3, 5, and 7 days post-inoculation. PI was calculated in relation to the control using the following equation proposed by Gotor-Vila et al. (2017): [(C-T) / C] x 100. Where C is the diameter of the controls and T that of the treatments. PI is expressed as the mean of the four replicates. P values were obtained from growth results using one-way analysis of variance (ANOVA), after confirmation of normality and equality of variances, and were contrasted using Tukey's *post hoc* test. Capital letters represent the differences between treatments with $P \leq 0.05$; $P \leq 0.01$; and $P \leq 0.001$, respectively. All statistical analyses were performed using SPSS 24.0 (IBM).

**F3 EXPERIMENT 1**		day 3						day 5						day 7					
		GROWTH (mm)		INHIBITION (%)	STATISTICS			GROWTH (mm)		INHIBITION (%)	STATISTICS			GROWTH (mm)		INHIBITION (%)	STATISTICS		
	TREATMENT	Mean	SD	Mean	P ≤ 0.05	P ≤ 0.01	P ≤ 0.001	Mean	SD	Mean	P ≤ 0.05	P ≤ 0.01	P ≤ 0.001	Mean	SD	Mean	P ≤ 0.05	P ≤ 0.01	P ≤ 0.001
**Double Dish Set**	CC	30.25	0.96	-	A	A	A	46.08	1.69	-	A	A	A	57.67	4.25	-	A	A	A
	F3/T34	19.00	2.19	37.19	BC	BC	BC	31.92	4.38	30.74	BC	BC	B	46.50	2.67	19.36	B	B	AB
	F3/T34-5.27	16.58	1.29	45.18	C	C	C	28.83	0.43	37.43	C	C	B	43.08	0.92	25.29	B	B	B
	F3/E20	22.08	1.17	27.00	B	B	B	35.25	1.10	23.51	B	B	B	48.42	1.66	16.04	B	B	AB
	F3/E20-5.7	18.50	1.60	38.84	C	BC	BC	32.25	1.52	30.02	BC	BC	B	41.67	4.51	27.75	B	B	B

		GROWTH (mm)		INHIBITION (%)	STATISTICS			GROWTH (mm)		INHIBITION (%)	STATISTICS			GROWTH (mm)		INHIBITION (%)	STATISTICS		
	TREATMENT	Mean	SD	Mean	P ≤ 0.05	P ≤ 0.01	P ≤ 0.001	Mean	SD	Mean	P ≤ 0.05	P ≤ 0.01	P ≤ 0.001	Mean	SD	Mean	P ≤ 0.05	P ≤ 0.01	P ≤ 0.001
**Non-Vented VOC Chaber**	CC	25.67	1.72	-	A	A	A	39.58	4.03	-	A	A	A	53.67	3.99	-	A	A	A
	F3/T34	24.00	0.98	6.49	A	AB	AB	38.25	0.88	3.37	A	A	AB	50.22	0.38	6.42	A	AB	A
	F3/T34-5.27	20.50	0.33	20.13	B	C	B	31.58	0.79	20.21	B	B	B	44.67	0.58	16.77	B	B	A
	F3/E20	25.42	0.63	0.97	A	A	A	38.42	0.96	2.95	A	A	AB	52.11	0.84	2.90	A	A	A
	F3/E20-5.7	21.58	0.83	15.91	B	BC	B	32.67	1.12	17.47	B	B	AB	44.33	0.58	17.39	B	B	A

		GROWTH (mm)		INHIBITION (%)	STATISTICS			GROWTH (mm)		INHIBITION (%)	STATISTICS			GROWTH (mm)		INHIBITION (%)	STATISTICS		
	TREATMENT	Mean	SD	Mean	P ≤ 0.05	P ≤ 0.01	P ≤ 0.001	Mean	SD	Mean	P ≤ 0.05	P ≤ 0.01	P ≤ 0.001	Mean	SD	Mean	P ≤ 0.05	P ≤ 0.01	P ≤ 0.001
**Vented VOC Chaber**	CC	26.25	0.57	-	C	C	C	37.67	0.88	-	C	B	BC	49.78	2.52	-	C	B	B
	F3/T34	28.92	0.79	-10.16	B	B	B	42.50	1.69	-12.83	B	A	AB	57.33	0.33	-15.18	B	A	A
	F3/T34-5.27	23.33	0.47	11.11	D	D	D	34.33	1.39	8.85	D	BC	C	44.42	2.11	10.77	D	C	B
	F3/E20	31.67	0.61	-20.63	A	A	A	46.08	1.26	-22.35	A	A	A	62.44	0.69	-25.45	A	A	A
	F3/E20-5.7	22.50	0.64	14.29	D	D	D	32.67	0.67	13.27	D	C	C	43.00	0.33	13.62	D	C	B

Supplementary table ST4. Experiment 2. Growth (mm) and Percentage of inhibition (PI) of *F. oxysporum* F3 confronted by *T. harzianum* T34, T34-5.27, E20, and E20-5.7 using DDS method (first row), Non-Vented VOC Chambers (second row), and Vented VOC Chambers (third row); after 3, 5, and 7 days post-inoculation. PI was calculated in relation to the control using the following equation proposed by Gotor-Vila et al. (2017): [(C-T) / C] x 100. Where C is the diameter of the controls and T that of the treatments. PI is expressed as the mean of the four replicates. P values were obtained from growth results using one-way analysis of variance (ANOVA), after confirmation of normality and equality of variances, and were contrasted using Tukey's *post hoc* test. Capital letters represent the differences between treatments with P ≤ 0.05; P ≤ 0.01; and P ≤ 0.001, respectively. All statistical analyses were performed using SPSS 24.0 (IBM).

F3 EXPERIMENT 2		day 3						day 5						day 7					
		GROWTH (mm)		INHIBITION (%)	STATISTICS			GROWTH (mm)		INHIBITION (%)	STATISTICS			GROWTH (mm)		INHIBITION (%)	STATISTICS		
	TREATMENT	Mean	SD	Mean	P ≤ 0.05	P ≤ 0.01	P ≤ 0.001	Mean	SD	Mean	P ≤ 0.05	P ≤ 0.01	P ≤ 0.001	Mean	SD	Mean	P ≤ 0.05	P ≤ 0.01	P ≤ 0.001
Double Dish Set	CC	26.17	1.29	-	A	A	A	41.08	0.88	-	A	A	A	53.67	2.16	-	A	A	A
	F3/T34	20.17	1.40	22.93	BC	BC	BC	30.92	1.87	24.75	B	B	B	42.75	3.14	20.34	B	BC	B
	F3/T34-5.27	16.83	0.64	35.67	D	D	C	26.33	1.44	35.90	C	C	B	37.42	1.13	30.28	C	C	B
	F3/E20	20.92	1.00	20.06	B	B	B	31.92	2.15	22.31	B	B	B	43.92	1.85	18.17	B	B	B
	F3/E20-5.7	17.83	0.96	31.85	CD	CD	BC	29.00	1.52	29.41	BC	BC	B	40.25	1.73	25.00	BC	BC	B

		GROWTH (mm)		INHIBITION (%)	STATISTICS			GROWTH (mm)		INHIBITION (%)	STATISTICS			GROWTH (mm)		INHIBITION (%)	STATISTICS		
	TREATMENT	Mean	SD	Mean	P ≤ 0.05	P ≤ 0.01	P ≤ 0.001	Mean	SD	Mean	P ≤ 0.05	P ≤ 0.01	P ≤ 0.001	Mean	SD	Mean	P ≤ 0.05	P ≤ 0.01	P ≤ 0.001
Non-Vented VOC Chaber	CC	25.42	0.57	-	A	A	A	38.42	1.20	-	A	A	A	52.75	1.26	-	A	A	A
	F3/T34	22.17	0.79	12.79	B	B	B	33.83	0.43	11.93	C	B	B	47.54	1.23	9.87	B	B	B
	F3/T34-5.27	18.25	0.17	28.20	C	C	C	28.83	0.43	24.95	E	C	C	41.00	0.33	22.27	C	C	C
	F3/E20	24.92	0.57	1.97	A	A	A	36.50	0.79	4.99	B	A	A	48.58	1.00	7.90	B	B	B
	F3/E20-5.7	19.42	0.57	23.61	C	C	C	30.58	0.50	20.39	D	C	C	43.17	0.58	18.17	C	C	C

		GROWTH (mm)		INHIBITION (%)	STATISTICS			GROWTH (mm)		INHIBITION (%)	STATISTICS			GROWTH (mm)		INHIBITION (%)	STATISTICS		
	TREATMENT	Mean	SD	Mean	P ≤ 0.05	P ≤ 0.01	P ≤ 0.001	Mean	SD	Mean	P ≤ 0.05	P ≤ 0.01	P ≤ 0.001	Mean	SD	Mean	P ≤ 0.05	P ≤ 0.01	P ≤ 0.001
Vented VOC Chaber	CC	24.25	0.50	-	B	B	B	38.75	0.74	-	B	B	A	52.58	2.28	-	B	B	A
	F3/T34	26.75	0.50	-10.31	A	A	AB	41.08	1.20	-6.02	AB	AB	A	54.83	1.55	-4.28	B	AB	A
	F3/T34-5.27	20.78	0.57	15.12	C	C	C	31.89	1.12	18.28	C	C	B	42.56	0.61	19.49	C	C	B
	F3/E20	27.67	0.27	-14.09	A	A	A	42.58	1.62	-9.89	A	A	A	58.67	1.00	-11.57	A	A	A
	F3/E20-5.7	21.17	1.29	12.71	C	C	C	32.42	1.83	16.34	C	C	B	44.50	2.01	15.37	C	C	B
